# Natural Language Processing Methods and Bipolar Disorder: Scoping Review

**DOI:** 10.2196/35928

**Published:** 2022-04-22

**Authors:** Daisy Harvey, Fiona Lobban, Paul Rayson, Aaron Warner, Steven Jones

**Affiliations:** 1 Spectrum Centre for Mental Health Research, Division of Health Research School of Health and Medicine Lancaster University Lancaster United Kingdom; 2 Department of Computing and Communications Lancaster University Lancaster United Kingdom

**Keywords:** bipolar disorder, mental health, mental illness, natural language processing, computational linguistics

## Abstract

**Background:**

Health researchers are increasingly using natural language processing (NLP) to study various mental health conditions using both social media and electronic health records (EHRs). There is currently no published synthesis that relates specifically to the use of NLP methods for bipolar disorder, and this scoping review was conducted to synthesize valuable insights that have been presented in the literature.

**Objective:**

This scoping review explored how NLP methods have been used in research to better understand bipolar disorder and identify opportunities for further use of these methods.

**Methods:**

A systematic, computerized search of index and free-text terms related to bipolar disorder and NLP was conducted using 5 databases and 1 anthology: MEDLINE, PsycINFO, Academic Search Ultimate, Scopus, Web of Science Core Collection, and the ACL Anthology.

**Results:**

Of 507 identified studies, a total of 35 (6.9%) studies met the inclusion criteria. A narrative synthesis was used to describe the data, and the studies were grouped into four objectives: prediction and classification (n=25), characterization of the language of bipolar disorder (n=13), use of EHRs to measure health outcomes (n=3), and use of EHRs for phenotyping (n=2). Ethical considerations were reported in 60% (21/35) of the studies.

**Conclusions:**

The current literature demonstrates how language analysis can be used to assist in and improve the provision of care for people living with bipolar disorder. Individuals with bipolar disorder and the medical community could benefit from research that uses NLP to investigate risk-taking, web-based services, social and occupational functioning, and the representation of gender in bipolar disorder populations on the web. Future research that implements NLP methods to study bipolar disorder should be governed by ethical principles, and any decisions regarding the collection and sharing of data sets should ultimately be made on a case-by-case basis, considering the risk to the data participants and whether their privacy can be ensured.

## Introduction

### Mental Health and Bipolar Disorder

In 2018, the Lancet Commission on global mental health and sustainable development reported that the global burden of disease related to mental health disorders has risen in all countries and that mental health services are frequently of a lower quality than those provided for physical health [1]. The 2013 Global Burden of Disease study [2] described depression as the predominant mental health problem worldwide, followed by anxiety, schizophrenia, and bipolar disorder, and the 2019 Global Burden of Disease study suggested that 1.2% (>815,000 cases) of the UK population has been diagnosed with bipolar disorder [3]. Bipolar disorder is a mood disorder associated with recurring episodes of extreme moods, ranging from severe depression to mania and with episodes lasting up to weeks at a time. Bipolar disorder has been shown to affect psychosocial functioning in areas of work, finance, cognition, and relationships [4], and people living with bipolar disorder are at a high risk for self-harm [5]. Of those diagnosed with bipolar disorder, 10%-20% will die by suicide, and therefore, the prevention of future episodes and the management of depressive and manic episodes are the major goals of treatment in bipolar disorder [6]. It is difficult to understand the lived experience of bipolar disorder through clinical practice alone, primarily because clinicians may only see their patients under a restricted set of conditions and those are likely to frame any discussion about the experiences of the patients.

The clinical diagnosis of bipolar disorder is a lengthy and costly process that takes an average of 9 years to complete [7]. A delayed diagnosis can have major implications for misdiagnosed individuals and may lead to inadequate or inappropriate treatments, a greater severity and frequency of mood episodes, and an increased risk for suicide among individuals who are later diagnosed with bipolar disorder [8]. Considering the economic implications, it is estimated that the total costs associated with bipolar disorder in the United Kingdom, including service costs and lost employment costs, could reach £8.2 billion (US $10.6 billion) by 2026 [9].

### Natural Language Processing and Bipolar Disorder

The World Health Organization states that health systems must do more to respond to the burden of mental health disorders and that many people living with mental illness do not receive the care that they need. The development of *strengthened information systems* to provide evidence for population health monitoring and mental health surveillance is 1 of the 4 major objectives of the World Health Organization Mental Health Action Plan 2013-2020 [10]. The Lancet Commission also stated that digital technology can be used both to provide support and tools to people living with mental illness and to facilitate the screening and diagnosis of mental disorders using big data approaches. The increasing use of social media combined with the computational infrastructure of health care systems in the advent of the maturation of natural language processing (NLP) and machine learning (ML) technologies [11] provides exciting possibilities to investigate large amounts of data at the population and individual level.

Le Glaz et al [12] explained how language plays an important role in mental health technologies and how NLP uses the language resources available to analyze text both qualitatively and quantitatively to provide deeper insights into these data. NLP methods can focus on various features, including lexical choices, syntax, and semantics, to perform tasks such as topic modeling, clustering, and classification. Le Glaz et al [12] described that NLP in mental health research comprises the following four main stages: (1) corpus creation—the most common corpora include electronic health records (EHRs), social media data (eg, Reddit and Twitter posts), and transcribed patient interviews; (2) corpus processing—extracting medical terms or processing blocks of language using specific searches; (3) classification methods—ML techniques including deep learning; and (4) goal—the ultimate goal of validating a hypothesis or studying the behavior of a specific population.

Mental health research related to bipolar disorder can benefit from NLP methods in several ways. First, large amounts of longitudinal data from health records can be analyzed to provide population-level insights and to contribute to the creation of semiautomated systems, for example, to improve the specificity and speed of diagnosis [13,14]. Second, NLP methods can also be used for more fine-grained analyses at an individual level by analyzing lived experience accounts of bipolar disorder. This could include monitoring the sentiment and effect of web-based interactions over time [15], using textual cues in web-based communication to shed light on language features that relate to a bipolar disorder diagnosis [16], or using emotion detection methods to learn more about how emotions fluctuate over time [17]. Using NLP methods in the study of bipolar disorder could contribute to greater personalization of care through in-depth analysis of large amounts of textual data [18] and may yield insights that would be difficult to obtain in a formal health care setting owing to financial and time constraints. Analyzing the language used in nonclinical settings also provides an opportunity to learn more about what people with bipolar disorder say unprompted in situations that are not framed by clinicians or researchers. Becker et al [19] suggested that there is a need for a common language between the data science community and the health care community. This common language would enable data scientists to understand the technologies that are needed and how these can be implemented with clients, and enable health care workers to understand technical capabilities and the type of data that is most useful in developing automated systems. Carr [20] also explained that patient and public involvement and the incorporation of knowledge of domain experts (such as people with personal experience of bipolar disorder) are vital for ethical decision-making, because it enables a more robust understanding of language and context.

### Objectives

To understand how NLP methodologies have been used to better understand bipolar disorder, we conducted a scoping review. Scoping reviews enable the researcher to present an overview of a diverse body of literature and allow for the synthesis of a range of study designs and methodologies without narrowing it down to a focused research question as in a systematic review [21]. The goal of this scoping review is in line with the definition by Daudt et al [22], which states that a scoping review aims “to map the literature on a particular topic or research area and provide an opportunity to identify key concepts; gaps in the research; and types and sources of evidence to inform practice, policy making, and research.”

An initial broad search of the published literature suggested that no previous studies have systematically reviewed the literature describing how NLP has been used to better understand bipolar disorder. Although there are reviews that have focused on the use of ML methods or big data in the study of bipolar disorder [23,24] or the use of ML and NLP for mental health more widely [12,25,26], this scoping review focused specifically on bipolar disorder and the application of NLP methods to this condition.

This scoping review explored how NLP methods have been used in research to better understand bipolar disorder and also to identify which aspects of bipolar disorder are underresearched and could be aided by computational linguistic methods (a definition of terms can be found in Multimedia Appendix 1 [27,28].

The four research questions that were used to guide this scoping review were as follows:

1. What trends can be observed in literature? (eg, What does the literature talk about? Where are the data sourced from?)
2. Which NLP methods have been used in the literature?
3. What are the clinical and practical applications reported in the literature?
4. What ethical considerations are present in the literature?

## Methods

### Overview

This scoping review was conducted with reference to the framework proposed by Arksey and O’Malley [29], expanded by Levac et al [30] and Daudt et al [22], and was informed by the guidance provided in the Joanna Briggs Institute (JBI) manual for evidence synthesis in scoping reviews [31]. This scoping review has been reported according to the PRISMA-ScR (Preferred Reporting Items for Systematic Reviews and Meta-Analyses extension for Scoping Reviews) checklist [32] and was also informed by the guidance provided in the JBI manual for evidence synthesis in scoping reviews [33].

### Search Strategy

A systematic and computerized search was conducted using 5 databases and 1 anthology: MEDLINE, PsycINFO, Academic Search Ultimate, Scopus, Web of Science Core Collection, and the ACL Anthology. The search was conducted between January 25, 2021, and August 27, 2021, and the search strategy was developed with informed advice from a topic librarian. There were no restrictions on the date of publication.

The search strategy used index terms and free-text terms to cover two core themes: (1) bipolar disorder and (2) NLP. Adjacency operators were used when incorporating free-text terms to ensure the specificity of the returned results. The full search terms are shown in Figure S1 in Multimedia Appendix 2 [13-17,34-66].

The final list of studies that were eligible for screening was imported into the Mendeley Reference Manager for duplicate removal before it was uploaded to Covidence [67], which was used for abstract and full-text screening and data extraction. Citation chaining was conducted on the final set of full-text papers used in this review.

### Inclusion and Exclusion Criteria

Only papers written in English and published as peer-reviewed papers, full-text workshops, or conference proceedings were included in this review. It should be noted that the need for a faster review process has made conference proceedings the dominant form of published research in Computer Science and NLP [68]. To be included, studies needed to explicitly describe the application of an NLP method to the study of bipolar disorder (including those studies in which bipolar disorder was one of multiple psychological disorders being studied, but only when the data for bipolar disorder were separable). Studies that described quantitative, qualitative, or mixed method designs were eligible for inclusion, and papers were only included if they described completed research. Study designs and protocols were excluded from the study.

Papers were excluded from the scoping review if they only included an abstract and if the methodology described ML, deep learning, or big data approaches that did not rely on language features, for example, using magnetic resonance imaging data for bipolar disorder classification. Systematic and scoping review papers were also excluded from this study.

### Study Selection and Screening Process

Initial screening of the titles and abstracts was conducted independently by the lead reviewer (DH) and the second reviewer (AW) using Covidence [67] to assess the suitability of the studies identified during the search for inclusion in the review. The eligibility criteria were tested in a pilot of 25 studies to ensure that the criteria were suitable for the review. The JBI [33] recommends that an agreement of 75% demonstrates that the inclusion and exclusion criteria performed well, and the agreement between the first and second reviewers for the pilot screening was 84%. After establishing that the eligibility criteria were valid, the reviewers screened the remaining papers independently and resolved any conflicts through discussion.

For all papers that passed the title and abstract screening, the lead reviewer located the full texts and screened them for eligibility in the review. The second reviewer (AW) screened 20.4% (23/113) of the papers at the full-text screening stage to verify their inclusion or exclusion, and a 100% agreement was achieved between the first and second reviewers. Data were extracted from the final papers that were eligible for inclusion by the first reviewer (DH) using a customized data extraction template that was designed and implemented in Covidence [67] and is shown in Table S5 in Multimedia Appendix 2. The data extraction template was piloted with 3 papers by the lead reviewer and was verified for accuracy by another member of the review team (PR). Minor changes were recommended for the data extraction template, including changing the phrasing of some fields and adding two fields that measured the reproducibility of computational linguistic papers—whether the authors released their code and data set with the paper. A reviewer (DH) extracted all data from the 35 included articles. The extracted data are available in Table S1 in Multimedia Appendix 2.

To enhance the transparency of the research process, as recommended by the JBI [33], the protocol for this scoping review has been registered with Figshare and is available for public access [69].

### Analysis

A narrative synthesis [70] of the included studies was undertaken to map the literature as outlined in the research questions. The data were presented using descriptive frequency tables and charts and summarized according to inductively developed objectives.

## Results

### Overview

**Figure 1:**
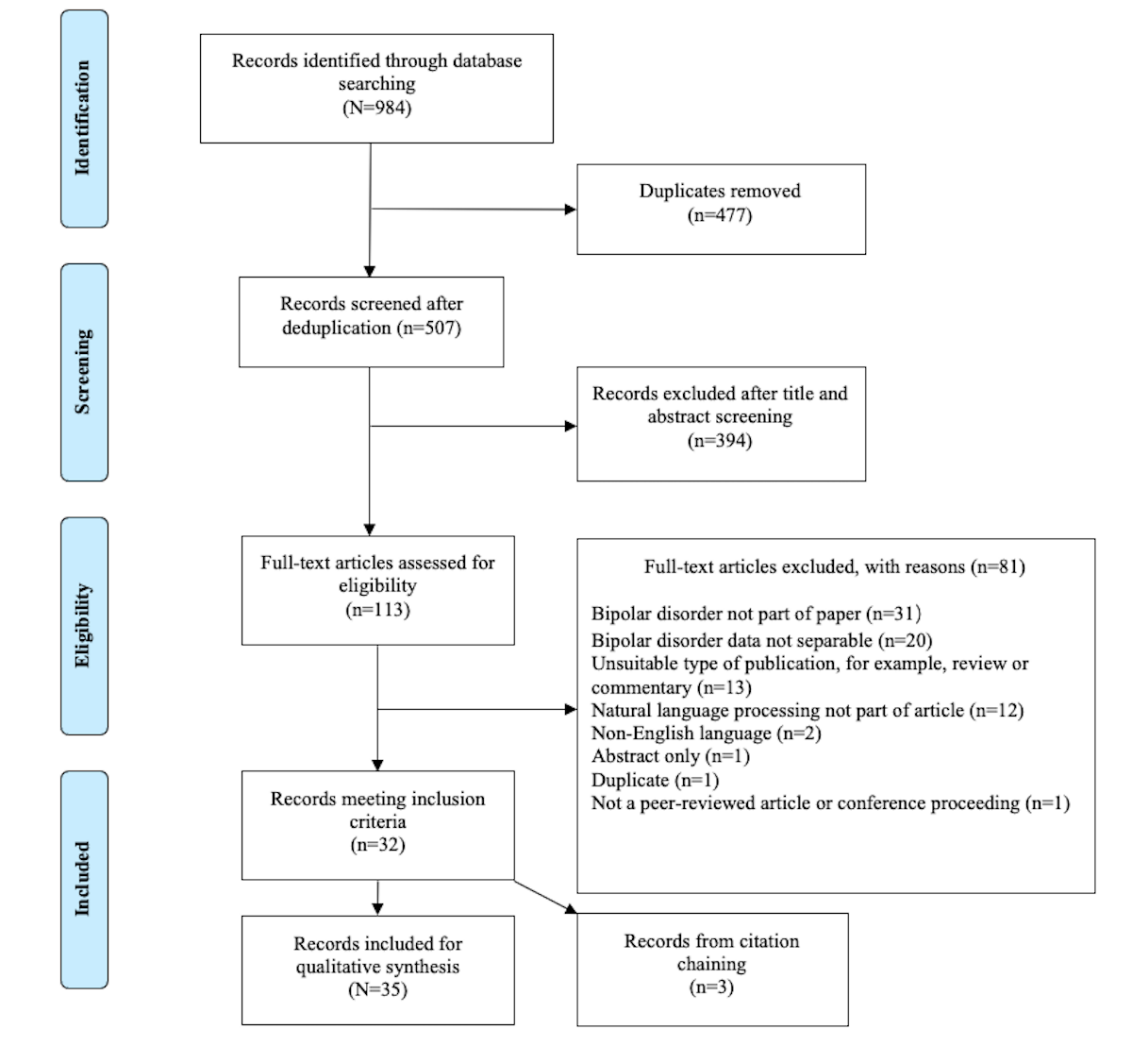
PRISMA (Preferred Reporting Items for Systematic Reviews and Meta-Analyses) flowchart of search history [71].

The initial search yielded 507 documents after deduplication. Of these, 394 (77.7%) were excluded after title and abstract screening because of ineligibility, leaving 113 (22.2%) for full-text review. After full-text screening, a further 81 (71.6%) articles were excluded. The reasons for the exclusions are shown in Figure 1. After full-text screening, 32 (91%) papers were included in the review, and 3 (9%) additional papers were included after citation screening of these papers, totaling 35 papers for inclusion in the scoping review. The results of the search and the study inclusion process are presented in the PRISMA (Preferred Reporting Items for Systematic Reviews and Meta-Analyses) flowchart in Figure 1 [71].

### Research Question 1: What Trends Can Be Observed in Research Which Uses NLP to Study Bipolar Disorder?

#### Publication Characteristics

This study identified 35 articles published in 25 different sources, including journals (17/35, 49%), workshops (11/35, 31%), and conference proceedings (7/35, 20%). The publication sources demonstrated the interdisciplinary nature of this type of research, with the studies presenting a crossover among the fields of health care, computational linguistics, and computer science. The most popular source for publication was the *Workshop on Computational Linguistics and Clinical Psychology,* where 6 (17%) of the articles were published. The remaining sources published ≤2 articles each and are detailed in Table S1 in Multimedia Appendix 2. Of the included articles, 97% (34/35) analyzed textual data in English, and 3% (1/35) used Norwegian data.

Table 1 shows the countries of publication represented by the location of the first authors, who were predominantly located in the United States (14/35, 40%), followed by the United Kingdom (7/35, 20%), Taiwan (5/35, 14%), and Australia (3/35, 8%).

In terms of the discipline of the first authors, 88% (31/35) of the articles were first authored by individuals in the fields of NLP, computer science, and bioinformatics (ie, computational fields), whereas only 9% (3/35) of the articles were first authored by individuals with a background in medicine or health care. Of these 3 articles, the disciplines of the first authors included psychiatry (n=2, 67%) and public health (n=1, 33%). There was also an article for which the discipline of the first author could not be confirmed, although the author was based at the Institute of Psychiatry, Psychology and Neuroscience, King’s College, London, when the article was published [57].

**Table 1:** The location of first authors (based on the location of affiliated institution).

Country (based on registered institution) of first author	Value, n (%)
United States	14 (40)
United Kingdom	7 (20)
Taiwan	5 (14)
Australia	3 (8)
Croatia	1 (3)
United States and Belgium and Germany	1 (3)
Germany	1 (3)
Korea and United States	1 (3)
Brazil	1 (3)
Korea	1 (3)

#### Data Source

**Figure 2:**
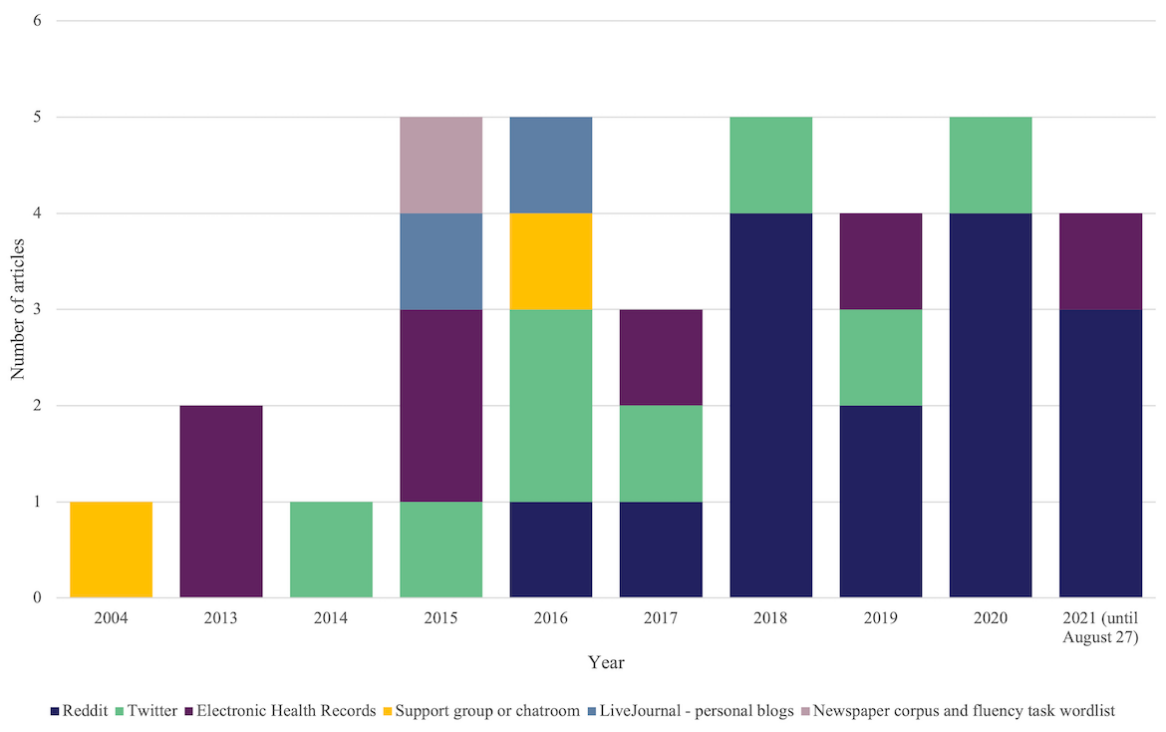
Number of studies published yearly by data source.

Figure 2 depicts the year-on-year trend in the publication of articles related to NLP and bipolar disorder. It is apparent that there has been an increase in the number of research articles related to this topic, from 1 relevant article in 2004 to 5 relevant articles in 2020. From 2015 onward, interest in this topic has remained fairly constant.

The published articles used a variety of sources for their corpora, including social media (Twitter, Reddit, support groups, chatrooms, and LiveJournal blogs), EHRs, and a newspaper corpus in conjunction with a fluency task wordlist. Figure 2 shows the increased use of social media since 2016, particularly after the publication of the study by Coppersmith et al [38] in 2014, which used Twitter to quantify mental health signals and could be described as a seminal work for this area of research. Since 2017, the only sources of data used in this field of research are Twitter, Reddit, and EHRs. The most commonly used data source for this type of research is Reddit (15/35, 43%), followed by Twitter (8/35, 23%) and EHRs (7/35, 20%). By including blogs, chatrooms, and support groups as a type of social media, 27 (77%) articles relied on data from social media, 7 (20%) used data from EHRs, and 1 (3%) used a newspaper corpus in conjunction with a fluency task wordlist.

#### Objectives of the Articles

**Figure 3:**
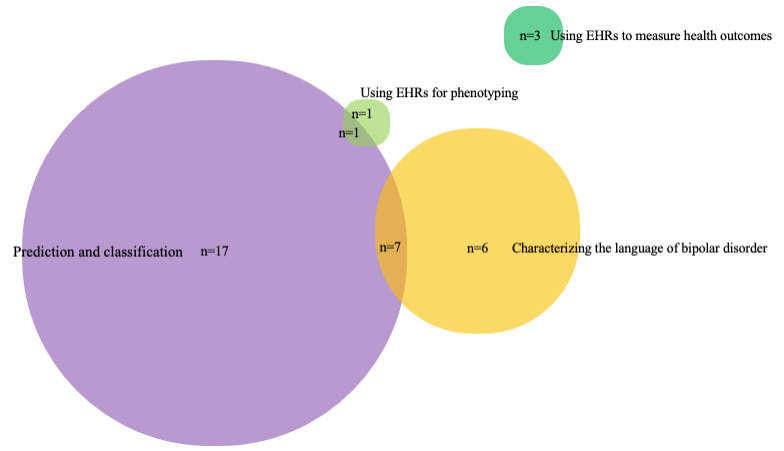
Grouped objectives of the studies. EHR: electronic health record.

The primary objectives of the articles were inductively categorized into four broad categories: (1) prediction and classification, (2) characterization of the language of bipolar disorder, (3) use of EHRs to measure health outcomes, and (4) use of EHRs for phenotyping. Figure 3 shows the number of articles that were grouped into each of these objectives and suggests that there is some overlap between these objectives. For example, Low et al [51] used Reddit data to characterize trends in health anxiety and to build a ML classifier that predicted mental health conditions.

Figure 3 suggests that the most prevalent objective was prediction or classification related to bipolar disorder and other mental health conditions, either from social media or using EHRs, and the second most frequent objective was to characterize the language of bipolar disorder and mental health. The 2 least common objectives were to use data from EHRs to measure health outcomes and for phenotyping.

### Research Question 2: Which NLP Methods Have Been Used in This Research?

Because of the broad variation in the specific aims of each article and the ever-increasing number of NLP tools available to researchers, there is large variation in the tools and methods that were used in the included papers. The following subsections group the articles using the aforementioned 4 objectives, describe the methods identified across the articles, and provide a qualitative summary of the results. Table S1 in Multimedia Appendix 2 provides more fine-grained details of the methods and results reported for each article in this review.

#### Prediction and Classification

The most frequent objective of the included articles was to use text from social media (n=22) [15-17,34,36-44,48,49, 51,54,59-63] or EHRs (n=3) [13,14,64] for prediction or classification purposes; for example, to predict a diagnosis of bipolar disorder based on features in the text. Among the 25 papers categorized into the objective of prediction and classification, 21 (84%) classified posts or users into a bipolar disorder class after comparison with a control group or with other mental health conditions. The aims of the remaining studies included; predicting the emotional tone in a bipolar disorder community, that is, how interactions in a web-based community affect people [15]; predicting the future occurrence of bipolar disorder based on a user's posts in a nonclinical subreddit before joining a bipolar disorder subreddit [63]; performing classification to measure subreddit uniqueness [40]; and using off-the-shelf algorithms to predict the demographic characteristics of people who self-reported a bipolar disorder diagnosis on Reddit [44]. There was large variation in the amount of data collected, with some authors reporting the number of relevant users and posts or comments and some reporting only the number of posts or users. The number of reported users within the bipolar disorder class varied from 50 patients with bipolar disorder listed in EHRs [14] to 19,685 Reddit users [44], and the number of reported posts or documents varied from 1000 blog posts [16] to >21 million Reddit posts [44].

Of the 22 studies that used social media for classification or prediction, 59% (13/22) verified a diagnosis. In the most rigorous cases, diagnoses were verified using detection patterns that incorporated diagnosis keywords collected from the corresponding Diagnostic and Statistical Manual of Mental Disorders (DSM), 5th Edition headings [37]. In other cases, regular expressions were used to pattern-match explicit expressions such as *I was diagnosed with bipolar* or to match mental health keywords used in bios. For the remaining articles (n=9, 41%), the authors used all posts collected from relevant bipolar disorder groups (eg, bipolar disorder portals on Twitter or subreddits related to bipolar disorder) without verifying whether the authors of these posts had received a diagnosis. The 3 studies that used EHRs to predict a diagnosis built their classifiers on a population of individuals within the health records who had received a previous diagnosis of bipolar disorder. The reliability of methods used to establish a diagnosis from social media data should be treated with some caution, because 9 of the articles within this review treated membership in a forum as confirmation of a diagnosis. In reality, forums are likely to include friends, family, and interested observers; therefore, this noisy verification of diagnosis could lead to unreliable data. Even when diagnoses are confirmed through more rigorous pattern matching using regular expressions, there is still a chance that users on the web may not have a genuine diagnosis. However, as described by Coppersmith et al [38], “Given the stigma often associated with mental illness, it seems unlikely users would tweet that they are diagnosed with a condition they do not have.”

Table S3 in Multimedia Appendix 2 shows the variety of ML models that have been applied to the objective of prediction and classification, and Table S4 in Multimedia Appendix 2 shows the different features that have been used as inputs for these models. The pooled data revealed that 19 of the articles used ML methods (see Multimedia Appendix 1 for defining terms) and 13 of the articles also implemented deep learning tools (many of the studies used a combination of both tools to compare the accuracy of different classifiers). Logistic regression was the most commonly used classifier for ML tasks, and convolutional neural networks were popular methods for studies that used deep learning. Several studies that implemented a deep learning methodology (Multimedia Appendix 1) also reported the use of an attention mechanism within their models (n=6). Galassi et al [72] described that the attention mechanism is part of a neural architecture that is able to “dynamically highlight relevant features of the input data, which, in NLP, is typically a sequence of textual elements.” The papers in this review that incorporated an attention mechanism described improved performance when compared with baseline methods, because the attention weights were used to demonstrate the most important words or sentences within the text for making classification decisions.

The features used most frequently for classification were derived from Linguistic Inquiry and Word Count (LIWC) [73] (for features relating to emotion and psychological state) and Term Frequency Inverse Document Frequency (TF-IDF) vectors. Pattern of Life (PoL) analytic features were introduced by Coppersmith et al [38], and relay information about the patterns and behavioral tendencies of users measured by social interactions (eg, tweet rate and number of @mentions) and were implemented in 4 of the studies. For studies that relied on deep learning methodologies, a number of different types of word embeddings were used as inputs for the models, including those derived from Bidirectional Encoder Representations [74], Word2vec [75], and global vectors for word representation (GloVe) [76].

In terms of accuracy (reported as overall accuracy, precision, recall, *F*_1_ score, and area under the curve defined in Multimedia Appendix 1) of the studies that aimed to classify a population into a bipolar disorder class (n=21), the following studies reported the highest scores (at 90%/≥0.9). Chang et al [36] reported a precision of 0.96 by using a random forest classifier based on TF-IDF features of Twitter users in single-task learning. Chen et al [17] reported an overall accuracy of 91.9% using the EMOTIVE ontology, LIWC, and Pattern of Life features for Twitter users with a logistic regression classifier in single-task learning. Huang et al [42] reported 95% precision for the female class using a pattern attention mechanism in single-task learning. Jiang et al [48] reported an *F*_1_ score of 0.982 for Reddit users using a Retrieval Augmented Language Model in single-task learning. Kim et al [49] achieved an overall accuracy of 90.2% for Reddit users using a convolutional neural network model with TF-IDF Word2vec vectors for single-task learning. Saravia et al [60] achieved a score of 96% precision for classifying Twitter users as having bipolar disorder using TF-IDF features with a random forest classifier in single-task learning, and Castro et al [13] reported an area under the curve of 0.93 for classifying an individual as having bipolar disorder or not using a logistic regression classifier in single-task learning from EHR data.

There were 4 articles, which used NLP methods for alternative classification purposes. Silveira et al [15] predicted how the emotional states of Reddit users would change after interacting on social media and framed this as a regression task that outperformed the baseline by a score of at least 12.9. Their results showed that general emotional states improved after interacting on the web and that the emotional tone of the final post by the thread author was generally more positive than their initial post. Gkotsis et al [40] used an ML classifier to measure the vocabulary uniqueness between mental health subreddits and demonstrated that there was a shared vocabulary across 3 different bipolar disorder subreddits. Thorstad and Wolff [63] demonstrated that future mental disorders could be predicted with an *F*_1_ score of 0.37 (which, although low, is above chance). Their work described the possibility of building classifiers to identify people at risk for developing mental illnesses. Finally, Jagfeld et al [44] used hybrid models to predict age and gender, which achieved 99% and 97% accuracies on their test set, respectively, as well as an inference model for location that achieved 78.4% test set accuracy.

The literature reports a number of successes that have been achieved in a variety of NLP prediction tasks related to bipolar disorder, and the heterogeneity in the methods of the papers, their data sets, and their individual objectives reflects the wide breadth of the field and the potential for this area of research. The results of each study are provided in Tables S1-S3 in Multimedia Appendix 2 with more details on the methods and tools used.

#### Characterizing the Language of Bipolar Disorder

A total of 13 papers were grouped into the objective of characterizing the language of bipolar disorder [16,35,37,39,40,42,44, 50,51,53,55,58,66] and used methods to build a more fine-grained picture of the linguistic behaviors of people living with bipolar disorder. Table S1 in Multimedia Appendix 2 provides more information on the focus, method, and main outcomes of each of the papers included in this category. The main patterns that emerged from this synthesis are described here.

LIWC was used by a number of authors to characterize language [16,37,39,40,50,66]. Cohan et al [37] found that bipolar disorder populations were significantly more likely to use first-person singular pronouns than their control group of Reddit users without a self-reported diagnosis, which they have suggested correlates with the LIWC category of authenticity. Gkotsis et al [40] also found a large number of first-person pronouns when comparing one of the bipolar subreddits (r/bipolarSOs) with other mental health groups within their study. The authors reported that this observation has been found in previous research on the language of depression and also touched on the idea of authenticity by suggesting that people with bipolar disorder may talk about personal issues more sincerely, which may increase their use of personal pronouns.

In all, 2 papers reported that the bipolar disorder community was more likely to talk about topics in the LIWC category of *Health* than a control group of Twitter users [39] and other web-based depression communities on LiveJournal blogs [16]. Yoo et al [66] identified clusters within the bipolar disorder community that were related to emotion and negative feelings, and their LIWC analysis also showed greater use of negative expressions when compared with people who posted on a depression subreddit. Kramer et al [50] described that the use of first-person pronouns was positively correlated with negative emotion words and that the use of *you* was positively correlated with positive emotion words. Huang et al [42] also reported that when using their graph-based algorithm, negative emotions were frequently used by their bipolar disorder group [42] and described that there was a significant difference in the use of tense between men and women in that women tended to use the present tense *I am* and that men preferred the past tense *I was.* Coppersmith et al [39] also described that the use of auxiliary verbs was significant in bipolar disorder users when compared with their control group according to the LIWC analysis.

In terms of the use of web-based social media sites, Jagfeld et al [44] used out of the box NLP models to report that most users who self-reported a diagnosis of bipolar disorder fell into the 30-49 year age range (47.5% of their data set) and were more likely to be classified as female (52.2%). This is in contrast with the demographic information of the general US Reddit adult population, with 64% of population being composed of people between the ages of 18 and 29 years and 67% of the US Reddit users being men [77]. McDonald and Woodward-Kron [53] focused on member role change in web-based communities using corpus methods and showed that users became like veterans the longer they used a web-based forum, dispensing advice using modal declaratives such as *You should consider seeing a professional*. Over the course of time, users preferred to describe themselves as *having bipolar* instead of *being bipolar*, and Kramer et al [50] reported in their study that users wrote more as they spent more time on the site. Park and Conway [55] assessed the readability of posts on Reddit over time and reported that although the posts of people posting on bipolar disorder subreddits were initially significantly more difficult to read than the control group, this improved as members participated more in the community. Rosenstein et al [58] conducted a verbal fluency task to understand how semantic structure is affected by bipolar disorder and discovered that people with bipolar disorder presented lower lexical diversity and semantic coherence than the control group.

Finally, 2 papers observed how external factors can influence the representation of bipolar disorder on the web. Low et al [51] used topic modeling and sentiment analysis to compare health-related anxiety presented on Reddit before the COVID-19 pandemic and during the pandemic. They demonstrated that the bipolar disorder subreddit did not seem to have suffered from induced health anxiety unlike other subreddits that were affected, such as those related to borderline personality disorder and posttraumatic stress disorder. They reported that there was no negative semantic change in the bipolar disorder subreddit by the middle of the pandemic, whereas other subreddits demonstrated significant negative semantic changes at this point. Budenz et al [35] used Twitter to collect tweets from communication spikes caused by external events (eg, the death of mental health advocate Carrie Fisher) to measure the amount of stigma or support presented in the communication. Their results showed that >67,393 (5.3% of the total sample) tweets discussed bipolar disorder, and 64.7% (4709/7281) of the bipolar disorder tweets that displayed stigma or support showed stigmatizing language. This was in contrast with 4.3% (38,336/873,590) of the tweets related to mental health and mental illness more generally that displayed stigmatizing language.

#### Using EHRs to Measure Health Outcomes

There were 3 articles that were grouped into the category of using EHRs to measure health outcomes [56,57,65]. All 3 studies used the South London and Maudsley Clinical Record Interactive Search (CRIS, 2021) database between 2013 and 2019 to assess different health outcomes of people diagnosed with bipolar disorder.

Wu et al [65] used the database to investigate smoking prevalence and the factors that influence it in populations receiving mental health care. Using open-text fields with General Architecture for Text Engineering (GATE) [78], the authors created a CRIS-IE smoking application using a shallow parsing rule–based approach to keywords. The results of this study demonstrated that patients with schizophrenia and schizoaffective disorder had a higher smoking prevalence than those with bipolar disorder. Patel et al [56] used the CRIS to assess the impact of mood instability on the clinical outcomes of individuals receiving secondary mental health care. TextHunter [79] was used to extract documentation related to mood instability from unstructured free-text fields, and supervised learning was used to develop support vector machine applications that were combined to generate a binary variable of instability. The prevalence of instability within one month of clinical presentation was 22.6% in the bipolar disorder population compared with 12.1% in the overall sample. Finally, Ramu et al [57] extracted descriptions of insight from text fields to determine whether poor insight recorded early after clinical presentation could predict subsequent service use. The authors used TextHunter [79] to create an ML algorithm based on a sample from clinical records to predict good or poor insight or to classify a document as irrelevant. The algorithm identified 61 patients with bipolar disorder who had at least one recording of poor insight, and the authors reported that a higher number of hospitalization episodes, unique antipsychotics, and inpatient days were all significantly correlated with poor insight.

#### Using EHRs for Phenotyping

The final characteristic used to group papers in this review was the use of EHRs for phenotyping (n=2) [13,52], in which case phenotyping relates to the process of characterizing or determining the observable characteristics of an individual and can refer to anything from a common trait, such as height or hair color, to presence or absence of a disease [80].

Castro et al [13] performed EHR-based phenotyping of bipolar disorder using EHRs to extract diagnostic data and compared the validity of an NLP algorithm with diagnostic interviews conducted by clinicians. The performance of the NLP algorithm for classifying case and control patients was assessed against DSM-IV Structured Clinical Interview for DSM-IV Disorders gold standard interviews, and the algorithm scored a positive predictive value of 0.85. Lyalina et al [52] used EHRs to identify the signature of 3 neuropsychiatric illnesses and to elucidate their phenotypic boundaries. The authors used text mining to annotate notes with concepts from 22 clinically relevant ontologies after preprocessing and negation checking, and enriched concepts were identified by reducing the number of case and control notes to 1000 each. A Fisher exact test was used to measure the enrichment within the sample. Their results demonstrated that the symptoms related to enriched phenotypes of bipolar disorder include migraines, irritable bowel syndrome, sleep disorders, ulcers, and mania and that there is substantial phenotypic overlap between bipolar disorder and schizophrenia. It should be noted that although not eligible for inclusion in this scoping review based on the methodology used, Mota et al [81] presented evidence to suggest that despite often sharing psychotic symptoms such as hallucinations, hyperactivity, and aggressive behavior, schizophrenia and bipolar disorder can successfully be differentiated based on the analysis of dream graphs, but psychometric scales cannot achieve the same result. Their work could provide a framework that uses behavioral biomarkers to drive a more objective, bottom-up search for anatomical and physiological biomarkers [81].

### Research Question 3: What Are the Clinical and Practical Applications of the Current Research?

It is important to understand why NLP methods have been applied to the study of mental health conditions and if this type of research is grounded in real-life implementations, particularly when large amounts of potentially sensitive social media data have been used.

The articles used in this review cited various reasons that make this type of study clinically relevant. Many authors have suggested that applying NLP methods to social media data could aid clinicians in their evaluations of bipolar disorder and that improved suicide prevention methods could be designed by combining ML methods and the medical community [34,39]. Sekulić et al [61] stated that the high incidence of suicide in bipolar disorder demonstrates the importance of early detection, and many authors suggested that applying NLP methods to social media data could contribute to the understanding of bipolar disorder and its detection and diagnosis [36,37,48,49,53,54,62,63]. Coppersmith et al [38] also suggested that using social media for large-scale data collection could complement existing methods and potentially make individual and population analyses quicker and cheaper. A number of the authors described that building a varied representation of bipolar disorder (eg, using features such as semantic deficit or attention weights) could provide a better understanding of the user experience, aid in diagnosis [16,42,58,64,66], and generate hypotheses for the clinical settings that may inform the provisioning of appropriate therapeutic resources [51].

Another practical application cited by the authors was the implementation of intervention systems based on flagging social media data for the moderator's attention [15,41,43]. Chen et al [17] and Park and Conway [55] described how different linguistic features can show how mental health conditions fluctuate over time and how these could help to identify worsening mental health. Gkotsis et al [40] suggested that urgency markers could be implemented for targeted interventions. Saha et al [59] reported that NLP methods could be used to screen and monitor health groups, and Saravia et al [60] and Silveira et al [15] suggested that social media data could be used to assist in the potential distribution of treatment to populations that are difficult to reach through traditional approaches. Ethical questions related to invasion of privacy, particularly when referring to populations who may have undetected mental illnesses, are raised by these possible innovations. It must be questioned whether the collection of data from social media platforms from a possibly unsuspecting population is ethical and it is also unclear who would be responsible for such an intervention.

In terms of the relevance of social media itself to people living with bipolar disorder, Kramer et al [50] described the hypothesis that 24 hour access to other people living with the same problem could reduce social isolation, improve coping skills, and improve patient knowledge about their own condition. Jagfeld et al [44] suggested that being aware of the demographics of web-based communities may help clinicians in recommending forums to their clients. Budenz et al [35] also described that social media advocacy can increase the amount of social support for people living with bipolar disorder to minimize the stigmatizing content posted on the web.

Finally, considering the use of NLP and medical records, Castro et al [13] and Dai et al [14] described that specific and predictive diagnostic algorithms could be created to assist with the diagnosis and to improve accuracy, achieving results that are comparable with diagnostic interviews. Other authors demonstrated how data, extracted using NLP, could improve care management and demonstrated the need, for example, to screen for the presence of instability on a routine basis or improve the assessment of smoking behavior [52,56,57,65].

### Research Question 4: What Ethical Considerations Are Present in the Literature?

A total of 60% (21/35) of articles used for the review referenced ethical considerations, and 40% (14/35) did not reference any ethical decision-making or design. The ethical considerations that were implemented are shown in Table 2, and Table 3 describes how the authors managed the code and data set release. It is interesting to note that the papers published until 2016 included limited discussion regarding ethical considerations, with only 47% (7/15) of papers published between 2004 and 2016 acknowledging ethical decision-making. In these earlier papers, discussion was generally limited to short statements, such as *all collected data were publicly posted to Twitter between 2008 and 2015* [39] or clarification that ethics approval had been granted.

Ethical considerations became more frequent in papers published from 2017 onward, with 67% (14/21) papers published in 2017-2021 incorporating (generally much more robust) ethics statements. The increased focus on ethics correlates with the drive toward open science, and recent guidelines were implemented by scientific communities, such as the Association for Computational Linguistics, that require authors to upload a checklist for responsible NLP research alongside any paper submission [82] and to include a discussion about positive and negative societal impacts that could stem from the research.

Several articles in this review provided a more detailed discussion of ethical issues. Benton et al [34] suggested that NLP models could be overgeneralized or used to identify specific people, and Cohan et al [37] stated that risks to individuals as a consequence of social media research should always be considered. Various articles [38,55,60,61] described that mental health analyses must be approached sensitively and [55] also described the nature of Reddit and the throwaway accounts that can protect users from social discrimination. The studies by Jagfeld et al [44] and Thorstad and Wolff [63] both described the issue of dual use in which research can be misused to harm the public (eg, by insurance companies) and also suggested that a possible solution to violating user privacy would be to inform people that the casual comments they make on social media may be mined [63]. Finally, Huang et al [42] stated that the practical application of their proposed model would only be used if both health care practitioners and patients agreed to use it.

**Table 2:** Ethical considerations.^a^

Ethical considerations	Values, n (%)
None	14 (40)
All user information anonymized	10 (29)
Ethical approval granted by relevant institution	9 (26)
Excerpts from data paraphrased or not published	3 (9)
No private tweets or protected user accounts used	2 (6)
Models did not include user features	1 (3)
URLs and usernames containing sensitive information removed	1 (3)
Comply with data usage agreement	1 (3)
Detailed initial psychological evaluations were excluded in the interest of public privacy	1 (3)

^a^Note that n does not equal total sample of 35 papers as some papers appear across multiple rows.

**Table 3:** Data set and code release.

Code and data set release			Values, n (%)
**Data set**			
	Availability not referenced	15 (43)	
	Not provided for ethical reasons but potentially available on request	11 (31)	
	Link to dataset or code to scrape dataset provided	6 (17)	
	Faulty link provided	2 (6)	
	Partial access provided	1 (3)	
**Code**			
	Not released	27 (77)	
	Access provided	7 (20)	
	Available on request	1 (3)	

## Discussion

### Principal Findings

This scoping review highlights the heterogeneity in the existing research that has used NLP methods to study bipolar disorder. The review suggests that the literature has been produced predominantly in the United States and the United Kingdom (21/35, 60%) and that 66% (23/35) of the studies used Twitter or Reddit as a source of data. The studies were predominantly led by authors from the computational and informatics fields (31/35, 88%), with only 3 articles being first authored by a health care expert. The articles were grouped into four inductively developed objectives: (1) prediction and classification, (2) characterization of the language of bipolar disorder, (3) use of EHRs to measure health outcomes, and (4) using EHRs for phenotyping, with most of the articles using NLP methods for prediction and classification purposes. The review suggests that using NLP for the study of mental health and bipolar disorder specifically is a growing field and it seems to have been influenced by the study of Coppersmith et al [38] when they provided a framework for obtaining quantifiable data in mental health research using Twitter.

The range of technologies that have been applied in the field reflects the ever-increasing possibilities for conducting research on language, with the most recent articles mainly favoring deep learning methodologies and word embeddings. The results from the existing research are varied and promising and indicate the usefulness of NLP methods to aid in diagnosis, predict the emotional impact of web-based interactions, characterize the language used by people living with bipolar disorder, and use phenotypes to better assist in care management. The 13 articles that characterized the language of bipolar disorder provided evidence to suggest that there are some observable linguistic traits that can be identified in a population with bipolar disorder; for example, an increased use of both first-person pronouns and negative emotion expressions, which could be useful in providing a better representation of bipolar disorder and developing early detection or intervention systems.

### Future Research

There are 4 areas for further research that are proposed based on the results of this review. First, Sekulić et al [61] referred to the high incidence of suicide in bipolar disorder and suggested that early detection systems could be developed. The use of signposting systems that could flag at-risk users for moderator intervention also has been discussed by several authors included in this review. Considering that bipolar spectrum disorders are associated with significant disinhibition and poor judgment, which can lead to the commission of risky and dangerous behaviors [83], a key area of future research should be to look at how risky behaviors (not just suicide) are discussed. This study would help to better understand how people living with bipolar disorder can be supported by health care providers to facilitate and improve their quality of life. Examples of risk-taking behaviors referenced in the literature on bipolar disorder include binge eating, excessive drinking, gambling, self-injury, and risky spending [83].

Second, several articles described the potential benefits of using social media for people living with bipolar disorder (eg, becoming more informed about bipolar disorder [53], improved emotional state after web-based interactions [15], and improved readability scores over time [55]). Further research could be conducted on how people living with bipolar disorder can best be supported on the web, and specific evaluative frameworks could be implemented for this purpose [50].

Third, although gender was not discussed in detail in this review, there are some contradictory results with regard to the portrayal of gender on social media by people living with bipolar disorder. Although Cohan et al [37] proposed that their Reddit corpus may be gendered toward men (because of the large amount of references to women), Jagfeld et al [44] used predictive algorithms to suggest that more than half of their Reddit corpus comprises women and that feminine-gender–identifying people with a BD diagnosis seem to be more likely to use Reddit and disclose their diagnosis. This area of research was also touched upon by Huang et al [42] who built a set of gender-specific syntactic patterns for bipolar disorder recognition. Further computational linguistic research could be conducted to determine how gender is presented by social media users and whether this correlates with demographic statistics for the diagnosis of bipolar disorder. Future work may demonstrate if there are demographic groups, which are currently undersupported by health care services and are instead seeking help online.

Finally, impaired social and occupational functioning in bipolar disorder has been presented frequently in the wider literature, although a review of the range of functioning in bipolar disorder has demonstrated that 16% of individuals diagnosed with the condition function at a high level and that functioning with bipolar disorder may have been underestimated by some clinical measures [84]. This area has not yet been explored using NLP methods, which presents an opportunity to provide a more balanced perspective on the wide range of ways in which people live with bipolar experiences based on lived experience narratives that are free from the potential ceiling effect of some clinical measures.

### Ethics

It is crucial that ethical design underpins research that uses NLP to study bipolar disorder, because there are serious ethical concerns relating to data use and anonymization and the concept of dual use. Although the research community must uphold rigorous ethical standards for data collection and protection, researchers are simultaneously moving toward open science to ensure transparency of research practices and to enable easy access to the data from which important conclusions have been drawn. Therefore, researchers are now faced with a conflict between the objectives of the open science movement and the need to uphold data privacy. Dennis et al [85] described that privacy and open science are on a collision course. A number of ideas have been proposed to manage this conflict, although there is still no clearly defined solution [85-87]. The British Psychological Society stated that internet-mediated research should obtain valid consent when it “cannot be reasonably argued that online data can be considered ‘in the public domain’” and that any data disseminated through the research should maintain the anonymity of the author [88]. They also stated that research should maximize benefits and minimize harm (to the research participants), referring to the fourth main principle of the Code of Human Research Ethics [89]. Friedrich and Zesch [90] described that ethics should be integrated into any NLP project and that NLP researchers should be mindful of the implications of developing any language technology. Benton et al [91] provided guidelines for ethical research using social media data stating that all research should consider the benefits and risks involved from the outset, thus enabling the implementation of strategies to make research as risk averse as possible.

The literature included in this scoping review suggests that there is ambiguity around the best practice for the ethical design of NLP methodologies, with 40% (14/35) of the articles making no reference to ethical decision-making and a wide range of methodologies for the articles that do. Future research that implements NLP methods to study bipolar disorder should be governed by ethical principles, and researchers should be aware that the best intentions could still have potentially harmful consequences. Although researchers are likely to be governed by the principals of open science, any decisions regarding the collection and sharing of data sets should ultimately be made on a case-by-case basis with consideration for the risk to the data participants and ensuring their privacy.

### Limitations

Although this scoping review was conducted according to a scoping review methodology and a previous protocol, there were some limitations that are worth noting.

First, as described throughout the review, there was large variation in the way NLP methods were described and indexed, and so it is possible that some relevant articles were not included in this review through the terms used in the search query.

Second, the data were extracted by only 1 reviewer because of the relatively high number of studies identified in the data extraction phase. An attempt was made to ensure accurate extraction by using a verified and standardized extraction form; however, the data that were extracted and used within the scoping review were predominantly qualitative, so it is likely that there could be researcher bias.

Finally, this review was conducted in an area of research that is constantly growing and developing and therefore only provides a time-stamped representation of the field.

### Conclusions

This scoping review provided an overview of 35 papers that applied NLP methods to the study of bipolar disorder. The data indicate that there are increasing opportunities for interaction between the clinical and NLP communities, and existing research shows how the analysis of language can be used to assist with and improve the provision of care for people living with bipolar disorder. There are 4 areas in bipolar disorder research that have been identified that may benefit from NLP methods, including the study of risk-taking behaviors, the research and design of web-based support groups specific to bipolar disorder, the study of social and occupational functioning, and the study of gender representation in bipolar disorder populations on the web.

## Appendix

Multimedia Appendix 1 Definition of terms.

Multimedia Appendix 2 Extracted data, data extraction template, and search strategy.

## Abbreviations

CRIS: Clinical Record Interactive Search
DSM: Diagnostic and Statistical Manual of Mental Disorders
EHR: electronic health record
GloVe: global vectors for word representation
LIWC: Linguistic Inquiry and Word Count
ML: machine learning
NLP: natural language processing PRISMA: Preferred Reporting Items for Systematic Reviews and Meta-Analyses
PoL: Pattern of Life
PRISMA-ScR: Preferred Reporting Items for Systematic Reviews and Meta-Analyses extension for Scoping Reviews
TF-IDF: Term Frequency Inverse Document Frequency
