# Supplementary material for: Natural Language Processing Methods and Bipolar Disorder: Scoping Review
Source: JMIR Ment Health. 2022 Apr 22;9(4):e35928. doi: 10.2196/35928 (PMC9077496; doi:10.2196/35928)
Supplement: Multimedia Appendix 1 [file mental_v9i4e35928_app1.docx]

**Multimedia Appendix 1 - Definition of Terms**

**Computational Linguistics and Natural Language Processing:** The terms "computational linguistics" and Natural Language Processing are often used synonymously, although the two areas of research tend to differ in purpose. Whilst both fields of research use an overlapping set of methods and techniques, computational linguistic research tends to answer linguistic questions using computational tools, and NLP research tends to use computational methods to process language for applications that are more related to computer science.

**Machine Learning:** Machine learning is the study and construction of algorithms which can learn from and make predictions from data, and is closely related to computational statistics. Machine learning models can be employed for predictive analysis as well as to reveal hidden insights from trends in data [27].

**Deep Learning:** Deep learning is a branch of machine learning which uses artificial neural networks and machine learning algorithms that have more than one hidden layer [27].

**Accuracy Scores:** Precision is the proportion of predicted positive cases that are correctly Real Positives, recall (otherwise known as sensitivity) is the proportion of Real Positive cases that are correctly predicted positive, and F1 is a function of prediction and recall which can be useful to find a balance between the precision and recall values or where there are imbalanced classes in the dataset. The Area Under a ROC (receiver operating characteristic) Curve (AUC) measures the accuracy of a quantitative diagnostic test, where the ROC curve shows the relationship between sensitivity (precision) and specificity (inverse recall or the true negative rate) [28].
