# Supplementary material for: Natural Language Processing Methods and Bipolar Disorder: Scoping Review
Source: JMIR Ment Health. 2022 Apr 22;9(4):e35928. doi: 10.2196/35928 (PMC9077496; doi:10.2196/35928)
Supplement: Multimedia Appendix 2 [file mental_v9i4e35928_app2.docx]

**Multimedia Appendix 2 - Extracted Data, Data Extraction Template and Search Strategy**

Table S1. Data extracted from review articles

| **Multi-Task Learning for Mental Health using Social Media Text (Benton et al.)** [34] | | | | | | | | | | | | | | | | | |
| --- | --- | --- | --- | --- | --- | --- | --- | --- | --- | --- | --- | --- | --- | --- | --- | --- | --- |
| **First Author Discipline**  Natural Language Processing/ Computer science | **Study Context**  Anxiety, bipolar disorder, depression, panic disorder, eating disorder, PTSD, schizophrenia, suicide. | | **Diagnosis confirmed (where relevant) e.g. by self-report**  Self-reported diagnosis (through Tweets matching statements such as 'I have been diagnosed with CONDITION') | | **Data Source**  Twitter | | **Sample size**  234 users with only bipolar disorder (may be more who have other comorbidities), average 3521 tweets per user.  9611 users in total.   4820 control. | | **Clinical Relevance Proposed by Author**  Multi-task learning can aid clinicians in their evaluations and improve suicide prevention efforts. | | **Journal**  Proceedings of the 15th Conference of the EACL (2017) | | **Ethical Considerations**  Discussion about overgeneralization and using the model to single out people. To minimize this risk the authors do not provide a selection of features or representative examples. | | **Is the dataset publicly available?**  Not reported | | **Is the code available for download?**  Not reported |
| **Focus:** Detect suicide and mental health conditions | | | | | | | | | | | | | | | | | |
| **Method:** Multi-task learning for detecting suicide risk and mental health conditions. Neural multi-task architecture to share parameters across tasks, using various classifiers: logistic regression, multilayer perceptron single task learning model, neural multi-task learning model. Implemented in Keras, character n-grams used as input to model. | | | | | | | | | | | | | | | | | |
| **Key Findings:** Single task models do not perform as well as multi-task models. Prediction of conditions like bipolar disorder which have the least amount of data are significantly improved by the model also predicting comorbid conditions. MTL performs better likely due to increased feature set size. Gender helps predict mental health conditions but not significantly. | | | | | | | | | | | | | | | | | |
| Bipolar AUC:  LR 72 STL 55 MTL 74 MTL + gender 75 | | Bipolar TPR performance at FPR = 0.1 LR 0.24 STL 0.22 MTL 0.25 MTL+ gender 0.26 | |  | |  | |  | |  | |  | |  | |  | |
| **Objective (Annotated by reviewer through inductive analysis):** Prediction and classification | | | | | | | | | | | | | | | | | |

| **Mental illness and bipolar disorder on Twitter: implications for stigma and social support (Budenz et al.)** [35] | | | | | | | | | |
| --- | --- | --- | --- | --- | --- | --- | --- | --- | --- |
| **First Author Discipline**  Public health | **Study Context**  Mental health, mental illness and bipolar disorder. | **Diagnosis confirmed (where relevant) e.g. by self-report**  N/A | **Data Source**  Twitter | **Sample size**  6,712,854 tweets collected in total. Subsample for spikes included 1,270,902 tweets.  67,393 tweets specific to bipolar disorder in the subsample. | **Clinical Relevance Proposed by Author**  Opportunity for social media advocacy to reduce stigma and increase social support to people living with BD. | **Journal**  Journal of Mental Health | **Ethical Considerations**  Not discussed | **Is the dataset publicly available?**  Not reported | **Is the code available for download?**  Not reported |
| **Focus:** Quantify the distribution of stigma and social support messaging about Mental Health/ Illness and Bipolar Disorder on Twitter | | | | | | | | | |
| **Method:** The authors first built a classifier which measured whether Tweets from a sub-sample of communication spikes (N = 1,270,902) displayed stigma or support and retained relevant tweets for content analysis. | | | | | | | | | |
| **Key Findings:** 67,393 (5.3% of sub-sample Tweets) discussed bipolar disorder and 64.7% of the bipolar disorder tweets with stigma or support showed stigma (nonclinical/slang, minimizing, self-stigma), compared to 4.3% stigma in the Tweets related to mental health and mental illness which displayed stigma or support. | | | | | | | | | |
| **Objective (Annotated by reviewer through inductive analysis):** Characterizing the language of bipolar disorder^a^ | | | | | | | | | |

| **Validation of electronic health record phenotyping of bipolar disorder cases and controls (Castro et al.)** [13] | | | | | | | | | |
| --- | --- | --- | --- | --- | --- | --- | --- | --- | --- |
| **First Author Discipline**  Data science | **Study Context**  Bipolar disorder. | **Diagnosis confirmed (where relevant) e.g. by self-report**  Diagnosis from medical notes | **Data Source**  Electronic Health Records | **Sample size**  209 patients used to develop the algorithm.  21,869 total patients classified with bipolar disorder by all algorithms.  296,356 total controls. | **Clinical Relevance Proposed by Author**  Highly specific and predictive diagnostic algorithms can be developed using EHRs. | **Journal**  American Journal of Psychiatry | **Ethical Considerations**  The Partners HealthCare System institutional review board approved all aspects of this study. | **Is the dataset publicly available?**  Research Patient Data Registry (RPDR) - dataset available to faculty members, non-faculty must have a sponsor to use the dataset. | **Is the code available for download?**  Not reported |
| **Focus:** Validate the use of EHRs to diagnose bipolar disorder. | | | | | | | | | |
| **Method:**  Clinician reviewed features were used to train models to predict the probability of bipolar disorder diagnosis using adaptive LASSO procedure.  13 features relevant to bipolar disorder e.g. mention of anxiety disorder, mention of psychotic episode. | | | | | | | | | |
| **Key Findings:** Performance of the 95-NLP algorithm was assessed against a SCID-IV classification of the case and control studies identified by the algorithms. Positive predictive value was calculated as the proportion of cases diagnosed with bipolar disorder by SCID-IV given the algorithm diagnosis of bipolar disorder. According to SCID-IV gold standard interviews, the cases selected by 95-NLP had a PPV of 0.85, the highest out of all the algorithms used.   Final model for classifying each note as indicating a bipolar disorder diagnosis yielded AUC of 0.93. | | | | | | | | | |
| **Objective (Annotated by reviewer through inductive analysis):** Prediction and classification; Using EHRs for phenotyping | | | | | | | | | |

| **Subconscious Crowdsourcing: A feasible data collection mechanism for mental disorder detection on social media (Chang et al.)** [36] | | | | | | | | | |
| --- | --- | --- | --- | --- | --- | --- | --- | --- | --- |
| **First Author Discipline**  Information systems and applications | **Study Context**  Bipolar disorder and borderline personality disorder. | **Diagnosis confirmed (where relevant) e.g. by self-report**  Self-reported diagnosis through keyword matching in Twitter bio (e.g., borderline, bipolar, BD) | **Data Source**  Twitter | **Sample size**  Bipolar disorder patients: 278 users on bipolar disorder portal; 347,774 tweets.  Bipolar disorder experts: 9 users; 14,505 tweets.   Total case users: 539  Control: 548 random users and 796,957 tweets | **Clinical Relevance Proposed by Author**  Can help to understand mentally ill users adopting techniques such as community detection and network analysis. | **Journal**  2016 IEEE/ACM International Conference on Advances in Social Networks Analysis and Mining (ASONAM) | **Ethical Considerations**  Not discussed | **Is the dataset publicly available?**  Not reported | **Is the code available for download?**  Not reported |
| **Focus:** Sharing and collecting of latent data on social media to build predictive models for mental disorders. | | | | | | | | | |
| **Method:**  Feature extraction: 1.TFIDF unigrams and bigrams 2.LIWC features - only words related to psychological behavior 3.Pattern of Life features (emotional scores, age and gender prediction, polarity features, social features e.g. tweeting frequency, frequent mentions).  Separate random forest classifiers used to train models for the three features for each condition, using 10-fold cross validation. | | | | | | | | | |
| **Key Findings:**  Bipolar disorder average precision using 10 fold CV:  Pattern of life 0.91 LIWC 0.9 TFIDF 0.96 | | | | | | | | | |
| **Objective (Annotated by reviewer through inductive analysis):** Prediction and classification | | | | | | | | | |

| **Tweeting your mental health: Exploration of different classifiers and features with emotional signals in identifying mental health conditions (Chen et al.)** [17] | | | | | | | | | |
| --- | --- | --- | --- | --- | --- | --- | --- | --- | --- |
| **First Author Discipline**  Information management | **Study Context**  Bipolar disorder, depression, post-traumatic stress disorder and seasonal affective disorder. | **Diagnosis confirmed (where relevant) e.g. by self-report**  Self-reported diagnosis (using regular expression 'I was diagnosed with CONDITION'.) | **Data Source**  Twitter | **Sample size**  Total 1372 case users.  438 users with bipolar disorder, average of 1.5k Tweets per user.   6596 controls. | **Clinical Relevance Proposed by Author**  Mapping emotional features can show how mental health conditions change over time which could be incorporated into a therapeutic intervention and help people to become aware of their emotions. | **Journal**  Proceedings of the Annual Hawaii International Conference on System Sciences | **Ethical Considerations**  No private messages or protected user accounts were accessed by the researcher, and all collected tweets was publicly posted on Twitter. | **Is the dataset publicly available?**  Not reported | **Is the code available for download?**  Not reported |
| **Focus:** Use fine grained sentiment/emotion analysis for the detection of mental health conditions from social media data. | | | | | | | | | |
| **Method:** Three sets of features used to train various classifiers: LIWC, Pattern of Life (PoL) and fine-grained emotion features using EMOTIVE sentiment algorithm.  The experiments involved four separate binary classification tasks against the control (which was a separate set of Tweets scraped from one day of data on Twitter). Feature sets used in concatenated feature vectors for each user, classification evaluated through leave one out cross validation. Classifiers used: Logistic Regression, Support Vector Machine (SVM), Naive Bayes (NB), Decision Trees (DT), and Random Forests (RF). | | | | | | | | | |
| **Key Findings:** The EMOTIVE feature set shows better performance than LIWC and PoL when used separately, suggesting that they provide a more abstract emotional aspect.  Results indicate that the PoL features are less relevant for the bipolar disorder class, and the PoL features show little correlation with the other two feature sets. The best performing classifier for bipolar disorder condition was using all three feature sets together with a leave one out cross validation accuracy of 91.9%, with precision of 0.909 and recall of 0.897 (using logistic regression). | | | | | | | | | |
| **Objective (Annotated by reviewer through inductive analysis):** Prediction and classification | | | | | | | | | |

| **SMHD: A large-scale resource for exploring online language usage for multiple mental health conditions (Cohan et al.)** [37] | | | | | | | | | |
| --- | --- | --- | --- | --- | --- | --- | --- | --- | --- |
| **First Author Discipline**  Computer science and Natural Language Processing | **Study Context**  ADHD, anxiety, autism, bipolar disorder, depression, eating disorder, OCD, PTSD, schizophrenia | **Diagnosis confirmed (where relevant) e.g. by self-report**  Self-reported diagnosis (through pattern matching statements such as 'I have been diagnosed with CONDITION') | **Data Source**  Reddit | **Sample size**  Total 20,406 diagnosed users and 335,952 matched controls.  6434 self-reported diagnosed users with bipolar disorder, 575,000 posts, 157.6 posts per user. | **Clinical Relevance Proposed by Author**  Dataset can be used to develop methods to better identify mental health conditions through language. | **Journal**  Proceedings of the 27th International Conference on Computational Linguistics, pages 1485–1497 Santa Fe, New Mexico, USA, August 20-26, 2018. | **Ethical Considerations**  Discussion that the risk to individuals from social media research should always be considered, and that the risk from the SMHD dataset is minimal. No excerpts of the data are publicly posted, users were not contacted and there was no attempt to identify users. Usernames were anonymized. | **Is the dataset publicly available?**  Final dataset released with paper, available through a data usage agreement to protect the users' privacy. | **Is the code available for download?**  Not reported |
| **Focus:** Characterize linguistic style and psychological aspects of language | | | | | | | | | |
| **Method:**  LIWC is used to characterize linguistic style and psychological aspects of language. Binary and multi-class label classifiers are trained (either condition vs control or most likely condition out of all classes).  Classifiers: LR: Bag of words features extracted over all posts by all users in training set. Features weighted using TFIDF. XGBoost: Same features as LR SVM: TFIDF bag of words fastText: 100 epochs, dimension of hidden layer set to 100  Convolutional neural network: Model trained for each condition for 100 epochs, each user's posts truncated to 15, 000 tokens. Each token represented by fastText embeddings. | | | | | | | | | |
| **Key Findings:** Authenticity is more prominent among mental health users which correlates with use of more personal pronouns. People with bipolar disorder show significantly more female references which may point to a gendered corpus towards males talking about females.  SVM is the best performing algorithm for bipolar disorder, and fastText provides the best F1 score for a multi-label multi-class test setting.   Bipolar disorder scores:  LR: F1 34.58 XGBoost: F1 44.68 SVM: F1 51.64 Supervised fastText: F1 50.81 CNN: F1: 38.34 | | | | | | | | | |
| **Objective (Annotated by reviewer through inductive analysis):** Characterizing the language of bipolar disorder; Prediction and classification | | | | | | | | | |

| **Quantifying mental health signals in Twitter (Coppersmith et al.)** [38] | | | | | | | | | |
| --- | --- | --- | --- | --- | --- | --- | --- | --- | --- |
| **First Author Discipline**  Computer science and psycholinguistics | **Study Context**  Bipolar disorder, depression, PTSD, SAD. | **Diagnosis confirmed (where relevant) e.g. by self-report**  Self-reported diagnosis (through Tweets matching statements such as 'I have been diagnosed with CONDITION') | **Data Source**  Twitter | **Sample size**  1238 case users, 5728 control.  394 users self-reported bipolar disorder diagnosis, 992,000 tweets. | **Clinical Relevance Proposed by Author**  Data collection methods can potentially make individual and population analyses much quicker and cheaper. | **Journal**  Proceedings of the workshop on computational linguistics and clinical psychology: From linguistic signal to clinical reality | **Ethical Considerations**  The authors intend for their 'results to inform the necessary ethical discussion regarding the balance between the utility of such data and the privacy of mental health related information'. | **Is the dataset publicly available?**  Not reported | **Is the code available for download?**  Not reported |
| **Focus:** Differentiate users with different mental health conditions using classifiers, and show how language of disorder differs from control. | | | | | | | | | |
| **Method:** 3 different sets of features used: LIWC features, Pattern of Life analytics and 1 gram and 5-gram language models also used to model the likelihood of sequences. Used to train a log linear classifier in scikit-learn with leave one out cross validation. | | | | | | | | | |
| **Key Findings:** All analytics show some ability to separate the classes meaning that they are useful signals. Language models show superior performance to other analytics suggesting more signals present in language than captured by PoL and LIWC. Overall classification accuracy using ROC curves for bipolar disorder at 10 and 20% false alarm rates:  10% - 0.64 20% - 0.82 | | | | | | | | | |
| **Objective (Annotated by reviewer through inductive analysis):** Prediction and classification | | | | | | | | | |

| **From ADHD to SAD: Analyzing the Language of Mental Health on Twitter through Self-Reported Diagnoses (Coppersmith et al.)** [39] | | | | | | | | | |
| --- | --- | --- | --- | --- | --- | --- | --- | --- | --- |
| **First Author Discipline**  Computer science and psycholinguistics | **Study Context**  ADHD, anxiety, bipolar disorder, borderline, depression, eating disorder, OCD, PTSD, schizophrenia, Seasonal Affective Disorder | **Diagnosis confirmed (where relevant) e.g. by self-report**  Self-reported diagnosis (through Tweets matching statements such as 'I have been diagnosed with CONDITION') | **Data Source**  Twitter | **Sample size**  2,013 total case users, age and gender matched control per case.  188 confirmed users for bipolar disorder, 3383 median number of tweets per user, 720,000 total tweets. | **Clinical Relevance Proposed by Author**  The information encoded in social media can be used by data scientists and psychologists to advance the field. | **Journal**  Proceedings of the 2nd Workshop on Computational Linguistics and Clinical Psychology: From Linguistic Signal to Clinical Reality, pages 1–10, Denver, Colorado, June 5, 2015. | **Ethical Considerations**  No private tweets used and all use of data approved by Institutional Review Board (IRB). | **Is the dataset publicly available?**  Not reported | **Is the code available for download?**  Not reported |
| **Focus:** Characterize language of mental health conditions. | | | | | | | | | |
| **Method:** LIWC analysis of categories, CLM classifier of each diagnosis against control (which use sequences of characters as input), hierarchical agglomerative clustering using CLMs. | | | | | | | | | |
| **Key Findings:** Users with bipolar disorder evince the LIWC categories of FUNCT, AUXVERB, COGMECH, HEALTH and DEATH significantly more frequently than control users. Clustering shows that bipolar disorder is grouped together with PTSD, eating disorders, anxiety and depression. The CLM (character n gram language model) classifier achieves an accuracy of 63% for bipolar disorder with 10% false alarms. | | | | | | | | | |
| **Objective (Annotated by reviewer through inductive analysis):** Prediction and classification; Characterizing the language of bipolar disorder | | | | | | | | | |

| **Deep Learning-Based Natural Language Processing for Screening Psychiatric Patients. (Dai et al.)** [14] | | | | | | | | | |
| --- | --- | --- | --- | --- | --- | --- | --- | --- | --- |
| **First Author Discipline**  Computer science | **Study Context**  Major depressive disorder, schizophrenia, bipolar disorder, minor depressive disorder, dementia. | **Diagnosis confirmed (where relevant) e.g. by self-report**  Diagnosis confirmed by psychiatrists | **Data Source**  Electronic Health Records (Medical Database of National Taiwan University Hospital (NTUHIMD) | **Sample size**  50 patients with bipolar disorder, 500 patients in total sample. | **Clinical Relevance Proposed by Author**  This method could assist with patient screening and diagnosis in psychiatry, especially in the case where physicians are not familiar with psychiatric diagnosis. | **Journal**  Frontiers in psychiatry | **Ethical Considerations**  National Taiwan University Hospital Research Ethics Committee (NTUH-201610072RINA) approval. | **Is the dataset publicly available?**  Anyone wishing to access the dataset must email the author. | **Is the code available for download?**  Available on GitHub. https://github.com/ken19980727/PsychiatricPatientScreening |
| **Focus:** NLP classification model that facilitates psychiatrist's diagnoses of 5 common mental health disorders. | | | | | | | | | |
| **Method:** Multi-label classification problem, two methods used. Diagnosis predicted if the output was larger than the threshold of 0.5.  1.Problem transformation - 5 binary classification tasks and 5 corresponding classifiers 2. Feature dependency - One classifier that could predict between 5 different diagnoses.  5 different neural networks for the two methods were developed 1. BERT as a baseline because of its performance in biomedical tasks.  2, 3, 4. Text classification architectures - BoW, linear models, CNN 5. Hierarchical attention network (HAN) | | | | | | | | | |
| **Key Findings:** Models trained with feature dependency tended to deliver more optimal performance. For bipolar disorder specifically the highest achieving model was HAN w2v.  Performance of HAN w2v model for bipolar disorder based on feature dependency:  P 0.626 R 0.5 F 0.556 | | | | | | | | | |
| **Objective (Annotated by reviewer through inductive analysis):** Prediction and classification | | | | | | | | | |

| **The language of mental health problems in social media (Gkotsis et al.)** [40] | | | | | | | | | |
| --- | --- | --- | --- | --- | --- | --- | --- | --- | --- |
| **First Author Discipline**  Data science | **Study Context**  Anxiety, borderline personality disorder, bipolar disorder, addiction, self-harm, Asperger's, autism, alcoholism, depression, schizophrenia, suicide. | **Diagnosis confirmed (where relevant) e.g. by self-report**  No | **Data Source**  Reddit | **Sample size**  3 bipolar subreddits included:  r/BipolarReddit, 14, 954 posts and 151, 588 comments r/bipolarSOs: 814 posts, 4,623 comments r/bipolar: 25, 868 posts and 198, 408 comments  Total number of comments and posts: 3,969,382 | **Clinical Relevance Proposed by Author**  This work can pave the way for future work on classification and the assignment of urgency markers in communication. | **Journal**  Proceedings of the 3rd Workshop on Computational Linguistics and Clinical Psychology: From Linguistic Signal to Clinical Reality, pages 63–73, San Diego, California, June 16, 2016 | **Ethical Considerations**  Not discussed | **Is the dataset publicly available?**  Provided at: https://www.reddit.com/r/datasets/comments/  3mg812/  full_reddit_submission_corpus_now_available_200/ | **Is the code available for download?**  Provided at: https://github.com/gkotsis/reddit-mental-health |
| **Focus:** Study the language features that are characteristic of mental health conditions | | | | | | | | | |
| **Method:** Features used included: lexical, syntactic, uniqueness of vocabulary and the expression of sentiment and happiness. Two main tasks: (1) Word based classification to assess subreddit uniqueness which compared the vocabularies of two subreddits at a time. Uni and bigrams used as input for naive bayes classifier using a balanced dataset of 5k words from each subreddit. (2) Detecting sentiment and happiness using topic specific dictionaries LabMT and AFINN. Only for the posts and not the comments. | | | | | | | | | |
| **Key Findings:** BipolarSOs subreddit stands out for a number of features even though it is the smallest subreddit included: high number of first-person pronouns and definite articles, sentences seem to be more complex based on parse trees, long verb clauses.  Around 60% accuracy in classifying between the correct bipolar disorder subreddits, up to 90% with the other mental health subreddits e.g. bipolarSOs and opiates recovery - demonstrating that the language between bipolar disorder and opiates is quite distinct but the language between all of the bipolar disorder subreddits is similar. | | | | | | | | | |
| **Objective (Annotated by reviewer through inductive analysis):** Characterizing the language of bipolar disorder; Prediction and classification | | | | | | | | | |

| **Characterisation of mental health conditions in social media using Informed Deep Learning (Gkotsis et al.)** [41] | | | | | | | | | |
| --- | --- | --- | --- | --- | --- | --- | --- | --- | --- |
| **First Author Discipline**  Data science | **Study Context**  Anxiety, borderline personality disorder, bipolar disorder, addiction, self-harm, Asperger's, autism, alcoholism, depression, schizophrenia, suicide. | **Diagnosis confirmed (where relevant) e.g. by self-report**  No | **Data Source**  Reddit | **Sample size**  41,636 posts for bipolar disorder from 3 subreddits.  Total number of case posts: 538, 272  476,388 non-mental health posts (control) | **Clinical Relevance Proposed by Author**  This method could be a first step towards targeted interventions e.g. flagging messages for moderator attention. | **Journal**  Scientific Reports | **Ethical Considerations**  Not discussed | **Is the dataset publicly available?**  From reddit user https://redd.it/3mg812 | **Is the code available for download?**  Not reported |
| **Focus:** Automatically identify Reddit posts related to mental health using binary classification and then classify mental health posts by theme-based subreddit groupings (multiclass classification) using deep learning. | | | | | | | | | |
| **Method:** 11 mental health themes identified across the 16 subreddits included, bipolar disorder is one of these themes comprising 3 subreddits. Classification of reddit posts 1) classify post as mental health or non-mental health 2) classify mental health posts into one of 11 themes. 80-20 training test split used for all classifiers.  Word embeddings used as features. | | | | | | | | | |
| **Key Findings:** CNN based approach yielded best prediction. For binary classification of mental health/ non mental health the CNN achieves an accuracy of 91.08% where 52% of the overall posts are mental health. Bipolar disorder most commonly misclassified as depression showing that there is overlap in post content between conditions.   CNN bipolar disorder multi-class classification:  Precision: 0.77 Recall: 0.60 F: 0.67 | | | | | | | | | |
| **Objective (Annotated by reviewer through inductive analysis):** Prediction and classification | | | | | | | | | |

| **Leveraging Linguistic Characteristics for Bipolar Disorder Recognition with Gender Differences (Huang et al.)** [42] | | | | | | | | | |
| --- | --- | --- | --- | --- | --- | --- | --- | --- | --- |
| **First Author Discipline**  Information systems | **Study Context**  Bipolar disorder. | **Diagnosis confirmed (where relevant) e.g. by self-report**  Time sensitive self-reported diagnosis (where users report time specific information in their disclosure Tweets e.g., 'I was diagnosed with bipolar in June 2017') | **Data Source**  Twitter | **Sample size**  Bipolar disorder users, 349. 231 female 118 male  Control 2600 Female: 1.6k Male: 1k | **Clinical Relevance Proposed by Author**  This method could serve as an assistive tool for BD assessments. | **Journal**  DSHealth ’19: 2019 KDD Workshop on Applied Data Science for Healthcare, August 4 – 8, 2019, Anchorage, Alaska | **Ethical Considerations**  The proposed model would only be employed as an assistive tool, and only if healthcare practitioners and patients agree to use it (rather than offer a diagnosis). The personal information of users in this study was masked and de-identified to protect privacy. | **Is the dataset publicly available?**  The dataset for control users is provided but not the BD dataset https://www.kaggle.com/crowdflower/twitter-user-gender-classification. | **Is the code available for download?**  Not reported |
| **Focus:** Focus on the language based features of social media posts by bipolar disorder users. Aim to establish a set of gender-specific syntactic patterns. | | | | | | | | | |
| **Method:** Adapts a graph-based extraction algorithm from the emotion detection work of Saravia et al. (2018). By constructing a word relation graph, the hidden word relations are preserved to enrich the patterns in comparison to traditional lexicon-based approaches. Patterns are built separately for both genders and integrated by a pattern attention mechanism.   Two sets of tweets are mapped to a graph function and a syntactic pattern is then derived. These patterns are used as features to train a model to recognize whether or not a user is likely to have bipolar disorder or belongs to the control group. Feature boosting used to preserve more representative information. The pattern model is compared with traditional methods such as LIWC and TFIDF, as well as BERT embeddings. | | | | | | | | | |
| **Key Findings:** The patterns most frequently used by the BD group include 1st person singular pronoun 'I' and topical words that express negative emotions e.g. 'tired', 'trying'. More positive topical words used by control group and less use of 1st person singular.  Both male and female use 1st person singular, but females tend to use the present tense 'I am' whereas males tend to use the past tense 'I was'. T test showed significant differences between tense usage. The best performing classifier was the pattern attention mechanism, which achieved 95% precision for the female class and 88% for males. | | | | | | | | | |
| **Objective (Annotated by reviewer through inductive analysis):** Prediction and classification; Characterizing the language of bipolar disorder | | | | | | | | | |

| **Hierarchical neural model with attention mechanisms for the classification of social media text related to mental health (Ive et al.)** [43] | | | | | | | | | |
| --- | --- | --- | --- | --- | --- | --- | --- | --- | --- |
| **First Author Discipline**  Data science | **Study Context**  Anxiety, borderline personality disorder, bipolar disorder, addiction, self-harm, Asperger's, autism, alcoholism, depression, schizophrenia, suicide. | **Diagnosis confirmed (where relevant) e.g. by self-report**  No | **Data Source**  Reddit | **Sample size**  Data from [41]  41,636 posts for bipolar disorder from 3 subreddits.  Total number of case posts: 538, 272  476,388 non-mental health posts (control) | **Clinical Relevance Proposed by Author**  Classifying posts in this manner is the first step to targeted interventions e.g. redirecting posts which require moderator attention. | **Journal**  Proceedings of the Fifth Workshop on Computational Linguistics and Clinical Psychology: From Keyboard to Clinic, pages 69–77 New Orleans, Louisiana, June 5, 2018 | **Ethical Considerations**  Excerpts used in study were paraphrased. | **Is the dataset publicly available?**  Yes from Reddit [41] | **Is the code available for download?**  Not reported |
| **Focus:** Apply a hierarchical RNN architecture to the classification of social media posts to see if a sequence-based model is more beneficial than a CNN model, and which parts of a post are more important for the correct classification. | | | | | | | | | |
| **Method:** This architecture progressively builds a document representation from sentence representations, which in turn are composed of the representations of the words they contain. The document representations are directly used by the architecture to make classification decisions. Document level architecture implemented using Keras toolkit. RNN models compared to CNN based architecture. | | | | | | | | | |
| **Key Findings:** RNN attention model is highest performing in overall classification. The attention mechanism is capable of efficiently distinguishing words and sentences of a document relevant for classification decisions - the beginning and final sentence of a document are the most important.   RNN-att model scores for bipolar disorder:  PR 0.79 / R 0.68 / F 0.73 | | | | | | | | | |
| **Objective (Annotated by reviewer through inductive analysis):** Prediction and classification | | | | | | | | | |

| **Understanding who uses Reddit: Profiling individuals with a self-reported bipolar disorder diagnosis (Jagfeld et al.)** [44] | | | | | | | | | |
| --- | --- | --- | --- | --- | --- | --- | --- | --- | --- |
| **First Author Discipline**  Natural Language Processing | **Study Context**  Bipolar disorder. | **Diagnosis confirmed (where relevant) e.g. by self-report**  Self-reported diagnosis (using patterns to match diagnosis statements e.g. 'I was diagnosed with BD today') | **Data Source**  Reddit | **Sample size**  19,685 users who match a self-reported BD diagnosis.  21,407,595 posts. | **Clinical Relevance Proposed by Author**  Investigating the identity and demographics of people who post online is relevant for clinicians who may want to recommend certain online forums to clients and to clinical researchers interested in recruiting via Reddit. | **Journal**  The Seventh Workshop on Computational Linguistics and Clinical Psychology: Improving Access @NAACL 2021 | **Ethical Considerations**  Detailed discussion around consent and anonymity of Reddit users, and the potentially harmful uses of the dataset. User anonymity was protected and only paraphrased posts presented in research. Discussion around dual use.  Study approved by Lancaster University FHMREC. | **Is the dataset publicly available?**  Due to ethical concerns, dataset will only be shared upon request with a data usage agreement. | **Is the code available for download?**  The code and patterns to identify self-reported MH diagnoses, age, and gender are released: https://github.com/glorisonne/reddit_bd_user_characteristics |
| **Focus:** Estimates and discusses clinical, demographic and identity characteristics of Reddit users who self-report a BD diagnosis. | | | | | | | | | |
| **Method:** Several NLP methods applied and compared to identify users with a self-reported diagnosis of bipolar disorder, and infer clinical and demographic characteristics i.e., age, country of residence and identity (gender characteristics). Two methods were used to retrieve a user's age, self-report where available, or a language use neural network model which predicts age [45] (or a hybrid implementation of these methods). Country of residence was identified using a method which uses different features from text to infer posting country [46]. Three methods were implemented to detect binary gender - a neural network model which makes predictions based on username [47], self-report, and Tigunova's language use model [45] (or a hybrid implementation of these methods) . Two authors manually annotated random subsets of users to check the automated characteristics extraction. 100 random users were included in this test set. | | | | | | | | | |
| **Key Findings:**  Age: The Hybrid method achieves 99% test set accuracy and 68% coverage on the full dataset. Country: Harrigian's [46] method assigns a country estimate to every user with 78% test set accuracy. Gender: The Hybrid gender identification method achieves 97% test set accuracy, gender-labelling 72% of users. Hybrid method assigned feminine gender to slightly more than half of the Reddit users (52.2%) for which it ascribed a gender identity. This sharply contrasts with only 9% feminine vs. 41% masculine gender-performing usernames among Reddit users who post in the top 10K subreddits with most posts. Considering a majority of male Reddit users and sex-equal prevalence of the diagnosis, feminine-gender-identifying people with a BD diagnosis seem to be more likely to use Reddit and/or to disclose their diagnosis.  Reddit users with a self-reported BD diagnosis, who are on average 27.7 years old at their first post, seem to overwhelmingly live in the US, and are more likely to identify with the feminine gender. Insofar they deviate from general Reddit as well as epidemiological statistics and also from participants in clinical studies. Less reddit users with a self-reported BD diagnosis are 18-29, but there are more users between the age of 30-49 compared to average US Reddit users. | | | | | | | | | |
| **Objective (Annotated by reviewer through inductive analysis):** Prediction and classification; Characterizing the language of bipolar disorder | | | | | | | | | |

| **Detection of Mental Health from Reddit via Deep Contextualized Representations (Jiang et al.)** [48] | | | | | | | | | | | | | | | | | | |
| --- | --- | --- | --- | --- | --- | --- | --- | --- | --- | --- | --- | --- | --- | --- | --- | --- | --- | --- |
| **First Author Discipline**  Computer science | **Study Context**  Schizophrenia, borderline personality disorder, post-traumatic stress disorder, eating disorder, major depression disorder, general anxiety disorder and bipolar disorder. | | **Diagnosis confirmed (where relevant) e.g. by self-report**  Self-reported diagnosis (using regular expressions to search for self-identification of diagnoses) | **Data Source**  Reddit | | **Sample size**  5,819,000 posts collected for bipolar disorder, 11,186 users.  Total number of users collected across cases: 35,606   6420 bipolar users included in classification task.  Not clear how control group is created. | **Clinical Relevance Proposed by Author**  Method applicable to real-world diagnostic scenarios. | | | **Journal**  Proceedings of the 11th International Workshop on Health Text Mining and Information Analysis | | | | **Ethical Considerations**  Not discussed | | **Is the dataset publicly available?**  Not reported | **Is the code available for download?**  Not reported | |
| **Focus:** Automatic detection of psychiatric disorders from linguistic content of social media posts. | | | | | | | | | | | | | | | | | | |
| **Method:** Compare methods based on contextual representations e.g. BERT attention-based model to logistic regression based on LIWC features. Binary and multi-class classification performed. For binary classification, trials are used both with and without the mental health sensitive posts (one will rely more heavily on syntactic data) at both the user and post level - user level detects whether individual has diagnosis based on aggregate data. | | | | | | | | | | | | | | | | | | |
| **Key Findings:** LIWC results indicate that mental health groups significantly more likely to use 'I'.  Linguistic traits for mental health more easily recognized at the user level. | | | | | | | | | | | | | | | | | | |
| Binary classification at user level:  Highest performing bipolar clean BERT-ATT: F1 0.879 Highest performing bipolar unclean REALM: F1 0.982 | | Lowest performing bipolar clean LIWC: F1 0.692 Lowest performing bipolar unclean LIWC: F1 0.773 | | | Multiclass classification at post level: Bipolar F1 0.615, but overall micro F1 for all classes of 0.2175, just above baseline. | | |  |  | |  |  |  | |  | | |  |
| **Objective (Annotated by reviewer through inductive analysis):** Prediction and classification | | | | | | | | | | | | | | | | | | |

| **A deep learning model for detecting mental illness from user content on social media (Kim et al.)** [49] | | | | | | | | | |
| --- | --- | --- | --- | --- | --- | --- | --- | --- | --- |
| **First Author Discipline**  Information science | **Study Context**  Mental illness (depression, anxiety, bipolar disorder, borderline personality disorder, schizophrenia, autism) | **Diagnosis confirmed (where relevant) e.g. by self-report**  No | **Data Source**  Reddit | **Sample size**  14,372 users for bipolar class and 41, 493 posts  Total number posts: 488,469 | **Clinical Relevance Proposed by Author**  Users could be alerted before they have approached counselling centers. Online service providers could predict probability of different mental disorders with user consent. | **Journal**  Scientific Reports | **Ethical Considerations**  All user information anonymized. This study was approved by the Ethical Committee and Institutional Review Board of the Department of Applied Artificial Intelligence, Sungkyunkwan University (#H1AAI2020). | **Is the dataset publicly available?**  The dataset is available by request https://jina-kim.github.io/dataset/20srep-mental | **Is the code available for download?**  Code is not referenced with paper but the study states: Other information used in this study can be accessed from the corresponding author with the reasonable request. |
| **Focus:** Identify which mental illness a user's post belongs to using deep learning. | | | | | | | | | |
| **Method:** 6 binary classification models developed and SMOTE used to address imbalance. People who wrote in bipolar disorder subreddit classified as 'bipolar', all other users in dataset labelled as 'non-bipolar'. 80/20 split training and test, XGBoost and CNNs were compared. TFIDF vectors used for XGBoost and word2vec word embeddings used with CNN. | | | | | | | | | |
| **Key Findings:** CNN model showed overall higher accuracy than XGBoost model.  XGBoost overall accuracy: 85.53 class bipolar: f1 53.59 class non-bipolar: f1 91.43  CNN overall accuracy: 90.20 Class bipolar: precision 87.22, recall 38.02, F1 52.95  Class non-bipolar: precision 90.40, recall 99.05, F1 94.53 | | | | | | | | | |
| **Objective (Annotated by reviewer through inductive analysis):** Prediction and classification | | | | | | | | | |

| **Text Analysis as a Tool for Analyzing Conversation in Online Support Groups (Kramer et al.)** [50] | | | | | | | | | |
| --- | --- | --- | --- | --- | --- | --- | --- | --- | --- |
| **First Author Discipline**  Computer science | **Study Context**  Bipolar disorder. | **Diagnosis confirmed (where relevant) e.g. by self-report**  No | **Data Source**  Bipolar disorder support chatroom | **Sample size**  80,136 individual chat messages on 289 days by 267 different visitors. | **Clinical Relevance Proposed by Author**  Provide an approach which can evaluate the effectiveness of online support. | **Journal**  CHI EA '04: CHI '04 Extended Abstracts on Human Factors in Computing Systems | **Ethical Considerations**  Not discussed | **Is the dataset publicly available?**  Not reported | **Is the code available for download?**  Not reported |
| **Focus:** Characterize the nature of discourse on a bipolar disorder support group | | | | | | | | | |
| **Method:** Framework for a word count tool which works similarly to LIWC. Each line of the chat log is analyzed and each word in a document was compared against each word in user specified dictionaries. Word counts collapsed across day and across individuals - the result is a data file comprised of a line for each nickname for each day, a word count for each category and total words used per day per nickname.  Word categories came from two sources: 1 - A core set of terms related to bipolar disorder, e.g. treatment methods and coping strategies. Two individuals with expertise in BD verified the 9 categories, which included anger, anxiety, depressed, disability, disorders, illness, mania, medication, treatment.  2 - Some categories taken from LIWC e.g. Pronouns, negative emotions, positive emotions, cognitive processes, social processes. | | | | | | | | | |
| **Key Findings:** Compared to emotion discourse (talking and writing), BD discourse appears to be no more negative than other genres, but is less self-focused and more focused on social processes.  Pronoun use associated with emotional valence. 'I' was positively correlated with negative emotion words, and 'you' was positively correlated with positive emotion words.  Words per day increased slightly but significantly with each visit day per user. | | | | | | | | | |
| **Objective (Annotated by reviewer through inductive analysis):** Characterizing the language of bipolar disorder | | | | | | | | | |

| **Natural Language Processing Reveals Vulnerable Mental Health Support Groups and Heightened Health Anxiety on Reddit During COVID-19: Observational Study (Low et al.)** [51] | | | | | | | | | |
| --- | --- | --- | --- | --- | --- | --- | --- | --- | --- |
| **First Author Discipline**  Neurolinguistics and cognitive science | **Study Context**  14 interest categories: Addiction, alcoholism, ADHD, anxiety, autism, bipolar disorder, Borderline Personality Disorder, depression, health anxiety, loneliness, PTSD, schizophrenia, social anxiety, suicide. | **Diagnosis confirmed (where relevant) e.g. by self-report**  No | **Data Source**  Reddit | **Sample size**  r/BipolarReddit: Pre-pandemic: ~ 2500 posts Mid-pandemic: ~1000 posts  Total case posts: Pre-pandemic: ~170,000 Mid-pandemic: ~115,000 | **Clinical Relevance Proposed by Author**  Understanding more about the convergence of disorders can inform the blending of treatment approaches or identify at-risk populations. | **Journal**  Journal of medical Internet research | **Ethical Considerations**  Not discussed | **Is the dataset publicly available?**  https://osf.io/7peyq/ | **Is the code available for download?**  https://osf.io/7peyq/ |
| **Focus:** Characterize the impact of coronavirus on different mental health groups | | | | | | | | | |
| **Method:** Subreddits scraped using pushshift over 2 timeframes: pre-pandemic between Jan 2018 and 2019, and mid-pandemic, Jan-April 2020. Only the first post from each user was kept from each timeframe.   Features used from the posts included LIWC, sentiment analysis, basic word and syllable counts, punctuation, readability metrics, TFIDF n-grams, suicidality lexicons, economic stress, isolation, substance use, domestic stress, guns.   Binary classification was performed on each specific mental health subreddit vs. a control group from the remaining subreddits (2700 control posts per subreddit), 80-20 train test split.   Tasks performed: -Trend analysis across mental health subreddits in the mid pandemic dataset -Unsupervised clustering to observe how the cluster-characteristics changed pre-pandemic an mid-pandemic -Topic modeling to compare topics from pre- and mid- pandemic -Measuring similarity between subreddits over time to estimate which subreddits are becoming more similar to each other over time | | | | | | | | | |
| **Key Findings:** The bipolar disorder subreddit does not appear to have suffered from induced health anxiety unlike other subreddits like BPD and PTSD. No negative semantic change in the bipolar disorder subreddit by mid pandemic, whereas for subreddits like ADHD and anxiety, they were showing significant negative semantic change.   Unsupervised clustering - bipolar disorder reddit has second greatest representation in the medication cluster.  F1 score for the bipolar disorder classification model trained and tested on pre-pandemic data, highest achieving models were stochastic gradient descent linear classifier with L1 penalty SGD L1, and SVM - both achieved 0.811. This dropped to 0.753 when tested on the mid-pandemic test set.   Features that make it more likely bipolar disorder will be predicted (positive coefficient): bipolar, manic, mania, lithium, mood, episode, psychiatrist, hospit, LIWC money, med  Features that make it less likely bipolar disorder will be predicted (negative coefficient): adhd, addict, bpd, ptsd, LIWC ingestion, LIWC anxiety, LIWC work, automated readability index, LIWC future tense | | | | | | | | | |
| **Objective (Annotated by reviewer through inductive analysis):** Prediction and classification; Characterizing the language of bipolar disorder | | | | | | | | | |

| **Identifying phenotypic signatures of neuropsychiatric disorders from electronic medical records (Lyalina et al.)** [52] | | | | | | | | | |
| --- | --- | --- | --- | --- | --- | --- | --- | --- | --- |
| **First Author Discipline**  Bioinformatics | **Study Context**  Autism, bipolar disorder and schizophrenia | **Diagnosis confirmed (where relevant) e.g. by self-report**  Diagnosis confirmed through medical records | **Data Source**  Electronic Health Records | **Sample size**  5242 patients with bipolar disorder; 185,680 notes  Total patients 7104, total notes 238,042 | **Clinical Relevance Proposed by Author**  Knowing the associations between co-morbidities could assist in devising care management protocols. | **Journal**  Journal of the American Medical Informatics Association | **Ethical Considerations**  All data used in the study was deidentified. The PAMF Institutional Review Board approved the study. Detailed initial psychological evaluations were excluded in the interest of public privacy. | **Is the dataset publicly available?**  Not reported | **Is the code available for download?**  Not reported |
| **Focus:** To understand how medical record driven text mining could elucidate phenotypic boundaries of 3 neuropsychiatric illnesses. | | | | | | | | | |
| **Method:** Analyzed medical records of over 7000 patients at two facilities using an automated text-processing pipeline to annotate the clinical notes with Unified Medical Language System codes and then searched for enriched codes, and associations among codes, that were representative of the three disorders. | | | | | | | | | |
| **Key Findings:** The results related to the medications associated with each condition suggest that bipolar disorder is the most variable in its presentation, and that autism and schizophrenia each overlap with distinct aspects of bipolar disorder.  In terms of the phenotypes associated with each disorder, there is substantial phenotypic overlap between bipolar disorder and schizophrenia. Patients are more likely to abuse alcohol and suffer from chronic health problems such as obesity and hepatitis.  Enriched phenotypes in bipolar disorder include symptoms associated with depression and anxiety, such as migraines, irritable bowel syndrome, sleep disorders, and ulcers, as well as the core diagnostic criterion of bipolar disorder itself: mania. Patient level analysis shows that there is distinct overlap between schizophrenic and bipolar patients, whereas autistic patients form a more unique cluster. | | | | | | | | | |
| **Objective (Annotated by reviewer through inductive analysis):** Using EHRs for phenotyping | | | | | | | | | |

| **Member roles and identities in online support groups: Perspectives from corpus and systemic functional linguistics (McDonald & Woodward-Kron)** [53] | | | | | | | | | |
| --- | --- | --- | --- | --- | --- | --- | --- | --- | --- |
| **First Author Discipline**  Computational linguistics | **Study Context**  Bipolar disorder. | **Diagnosis confirmed (where relevant) e.g. by self-report**  No | **Data Source**  Online support group bipolar disorder | **Sample size**  5700 users (2014), 57,000 posts and 8.4 million words | **Clinical Relevance Proposed by Author**  Findings from online and offline healthcare could be integrated and diverse situations encountered by patients can be recognized. | **Journal**  Journal of Discourse and Communication | **Ethical Considerations**  Not discussed | **Is the dataset publicly available?**  Referenced in paper but not available at link http://interrogator.github.io/corpkit | **Is the code available for download?**  http://interrogator.github.io/corpkit |
| **Focus:** Corpus-based case study of language usage in a large online bipolar forum. | | | | | | | | | |
| **Method:** Interrogation of pre-processed corpus using Corpkit focusing on lexicogrammatical changes. Focus on member role change, analysis of transitivity choices, and exploration of different relational processes related to being bipolar/ having bipolar/ feeling bipolar at each stage of membership. | | | | | | | | | |
| **Key Findings:** Users take on the role of a veteran the longer that they use the forum, and dispense advice using modal declaratives e.g. 'You should consider seeing a professional'. The use of 'we/us' increases with length of time spent on the board which generalizes the experience of bipolar disorder. Jargon becomes more common over the course of membership which demonstrates familiarity with forum norms and conventions. Over the course of time 'having bipolar' comes to be more preferred than 'being bipolar'. | | | | | | | | | |
| **Objective (Annotated by reviewer through inductive analysis):** Characterizing the language of bipolar disorder | | | | | | | | | |

| **Classification of mental illnesses on social media using RoBERTa (Murarka et al.)** [54] | | | | | | | | | |
| --- | --- | --- | --- | --- | --- | --- | --- | --- | --- |
| **First Author Discipline**  Computer science | **Study Context**  Depression, anxiety, bipolar disorder, ADHD, PTSD. | **Diagnosis confirmed (where relevant) e.g. by self-report**  No | **Data Source**  Reddit | **Sample size**  14,681 total number of case posts.  3009 posts within bipolar class.  2478 control posts. | **Clinical Relevance Proposed by Author**  Method has the potential to assist in the diagnosis of various disorders. | **Journal**  Proceedings of the 12th International Workshop on Health Text Mining and Information Analysis | **Ethical Considerations**  The dataset was pre-processed to remove any URLs or usernames that could potentially contain sensitive information. | **Is the dataset publicly available?**  https://drive.google.com/drive/folders/11aW_fpXjA-O51uv3xYY3xj6NWGh1VYh_ | **Is the code available for download?**  Not reported |
| **Focus:** Classify five prominent mental illnesses by analyzing unstructured user data from Reddit. | | | | | | | | | |
| **Method:** Reddit API used to crawl titles and posts in subreddits which were assigned a corresponding class label. Topics including music, travel, India, politics, English, datasets, mathematics and science were used to crawl data for the control class. First multi-class model that uses a transformer based architecture such as RoBERTa to analyze people's emotions and psychology. LSTM, BERT used as baseline models to compare to RoBERTa, all using word embeddings as input to the model. | | | | | | | | | |
| **Key Findings:** The RoBERTa model outperforms the LSTM and BERT models in all categories. The posts offer more valuable information than the titles alone.  0.89 overall F1 score shows that the model will detect mental illness posts the majority of the time, meaning that people seeking help will not go unnoticed if the classifier was deployed in the real-world.   Results for bipolar class:  P R F1  bipolar, posts: 0.88 0.79 0.83 bipolar, titles: 0.58 0.63 0.60 bipolar, posts + titles: 0.88 0.83 0.86 | | | | | | | | | |
| **Objective (Annotated by reviewer through inductive analysis):** Prediction and classification | | | | | | | | | |

| **Differentiating sub-groups of online depression-related communities using textual cues (Nguyen et al.)** [16] | | | | | | | | | |
| --- | --- | --- | --- | --- | --- | --- | --- | --- | --- |
| **First Author Discipline**  Computer science | **Study Context**  5 groupings: depression, bipolar disorder, self-harm, grief and suicide. | **Diagnosis confirmed (where relevant) e.g. by self-report**  No | **Data Source**  LiveJournal - Personal blogs | **Sample size**  10,000 individual users in whole dataset over 24 online communities which form the 5 groups of interest.  Bipolar disorder subgroup formed from 7 communities, 1000 posts with roughly 142 posts from each of the 7 communities. | **Clinical Relevance Proposed by Author**  Data mining of online communities has the potential to shed light on the representation of mental health communities. This information could be applied to machine learning in psychiatric research. | **Journal**  International Conference on Web Information Systems Engineering 2015 | **Ethical Considerations**  Not discussed | **Is the dataset publicly available?**  Not reported | **Is the code available for download?**  Not reported |
| **Focus:** Understand the differences in language styles between depression-related communities | | | | | | | | | |
| **Method:** Two feature sets were extracted from the data; 1) topics using LDA, number of topics set to 50 and 2) language styles - the proportion of words associated with the psycholinguistic categories defined in LIWC. A classifier was used to distinguish between the posts made in depression communities from those made by other subgroups, and LASSO was used to perform classification and observe feature importance. | | | | | | | | | |
| **Key Findings:**  LDA:  The bipolar disorder subgroup commonly discussed topic 23 (related to medication, and specifically Seroquel, an atypical antipsychotic drug used in the treatment of bipolar disorder),and topic 28 (which is predominately about bipolar disorder itself).  Linguistic styles: The bipolar disorder communities had a strong component of health-related features, which mirrors their discussion of medication-related topics. Applying LASSO to the LIWC features confirms the dominance of features related to health, anger, family, and death for the bipolar disorder, self-harm, bereavement, and suicide subgroups respectively.  Accuracy in classifications of depression vs. bipolar disorder:  LIWC: 73.9 % TOPIC: 75% Combined: 77.6% | | | | | | | | | |
| **Objective (Annotated by reviewer through inductive analysis):** Prediction and classification; Characterizing the language of bipolar disorder | | | | | | | | | |

| **Harnessing Reddit to Understand the Written-Communication Challenges Experienced by Individuals With Mental Health Disorders: Analysis of Texts From Mental Health Communities (Park & Conway)** [55] | | | | | | | | | | | | | | | | |
| --- | --- | --- | --- | --- | --- | --- | --- | --- | --- | --- | --- | --- | --- | --- | --- | --- |
| **First Author Discipline**  Biomedical and health informatics | **Study Context**  Bipolar disorder, depression and schizophrenia. | **Diagnosis confirmed (where relevant) e.g. by self-report**  No | | **Data Source**  Reddit | **Sample size**  Total number of case posts: 695,071  146,328 posts from 5019 members in the r/bipolar subreddit.  Total number of control posts: 1,849,655 | | **Clinical Relevance Proposed by Author**  This method could be used to indicate when someone's mental health is worsening and could allow moderators to provide timely support. | | **Journal**  Journal of medical Internet research | **Ethical Considerations**  The research reported in this study was exempted from review by the University of Utah's institutional review board (IRB; ethics committee; IRB 00076188) under Exemption 2 as defined in US Federal Regulations 45 CFR 46.101(b). No user identifiable information was reported to protect user privacy. | | | | **Is the dataset publicly available?**  Not reported | **Is the code available for download?**  Not reported | |
| **Focus:** Understand the impact of mental disorder on written communication related to language impairment | | | | | | | | | | | | | | | | |
| **Method:** Readability of posts assessed using Fleisch Kincaid grade level, Simple Measure of Gobbledygook (SMOG) index, Gunning Fog index, and Linsear Write formula. The mean of all 4 metrics was calculated. (Authors assume that high readability means inarticulate language as they did not encounter highly sophisticated language in manual analysis). | | | | | | | | | | | | | | | | |
| **Key Findings:**  Posts of people with bipolar disorder were significantly more difficult to read than the control group with less lexical diversity, but this improved as members participated more in the community | | | | | | | | | | | | | | | | |
| Communication challenges results: Mean lexical diversity (SE) r/bipolar: 0.85 (0.001) Mean lexical diversity (SE) of a control group r/happy: 0.93 (0.001) | | | Mean (SE) readability 4 metrics r/bipolar: 8.58 (0.02) Mean (SE) readability 4 metrics r/happy: 7.11 (0.02) | | | Quality of language changes :  Slope of mean lexical diversity (SE) r/bipolar: 5.04 (0.48)  Slope of mean lexical diversity (SE) r/happy: 0.34 (0.23) | | Slope of mean readability (SE) 4 metrics r/bipolar: −0.14 (0.02) Slope of mean readability (SE) 4 metrics r/happy: −0.01 (0.01) | | |  |  |  | | |  |
| **Objective (Annotated by reviewer through inductive analysis):** Characterizing the language of bipolar disorder | | | | | | | | | | | | | | | | |

| **Mood instability is a common feature of mental health disorders and is associated with poor clinical outcomes (Patel et al.)** [56] | | | | | | | | | |
| --- | --- | --- | --- | --- | --- | --- | --- | --- | --- |
| **First Author Discipline**  Consultant psychiatrist and Health Data Research UK Fellow with natural language processing | **Study Context**  Psychotic, affective or personality disorder. | **Diagnosis confirmed (where relevant) e.g. by self-report**  Diagnosis confirmed through medical records | **Data Source**  Electronic Health Records - SLaM CRIS database | **Sample size**  27,704 total number of patients.  2691 patients with bipolar disorder included | **Clinical Relevance Proposed by Author**  Results suggest that mood instability should be given more attention by clinicians. | **Journal**  BMJ Open | **Ethical Considerations**  The CRIS data resource received ethical approval as an anonymized data set for secondary analyses from Oxfordshire REC C (Ref:08/H0606/71+5. | **Is the dataset publicly available?**  The data accessed by CRIS remain within an NHS firewall and governance is provided by a patient-led oversight committee. Subject to these conditions, data access is encouraged and those interested should contact RS (robert.stewart@kcl.ac.uk), CRIS academic lead. | **Is the code available for download?**  Not reported |
| **Focus:** The objective of this study was to assess the impact of mood instability on clinical outcomes in a large sample of people receiving secondary mental healthcare. | | | | | | | | | |
| **Method:** TextHunter used to extract documentation of mood instability from unstructured free text fields of clinical assessment and correspondence.  A supervised machine learning approach was then used to identify sentences containing the constructs of interest. Further sentences were annotated to create training data for three bag of words SVM applications to identify mood instability, affective instability and emotional instability.  Applications were then applied to the case register and output was combined to generate a binary variable for each patient defined as documentation of instability within 1 month of presentation to SLaM. | | | | | | | | | |
| **Key Findings:** The overall prevalence in the sample of recorded mood instability within 1 month of clinical presentation was 12.1%, and 22.6% for bipolar disorder. The strongest diagnostic association of mood instability was seen among those presenting with bipolar disorder. This finding corroborates previous research which has indicated that mood instability is a key factor in bipolar disorder, distinct from episodes of mania and depression. Mood instability was associated with a greater number of days spent in hospital, a greater likelihood of compulsory admission to hospital and increased frequency of hospital admission up to 5 years following clinical presentation. Mood instability was also associated with an increased risk of antipsychotic prescription and non-antipsychotic mood stabilizer prescription. The data supported the hypothesis that mood instability is associated with poorer clinical outcomes and increased use of healthcare services. | | | | | | | | | |
| **Objective (Annotated by reviewer through inductive analysis):** Using EHRs to measure health outcomes^a^ | | | | | | | | | |

| **Recorded poor insight as a predictor of service use outcomes: cohort study of patients with first-episode psychosis in a large mental healthcare database (Ramu et al.)** [57] | | | | | | | | | |
| --- | --- | --- | --- | --- | --- | --- | --- | --- | --- |
| **First Author Discipline**  First author discipline unknown, but based at Institute of Psychiatry, Psychology and Neuroscience, King’s College London, London, UK | **Study Context**  First episode psychosis. | **Diagnosis confirmed (where relevant) e.g. by self-report**  Diagnosis confirmed through medical records | **Data Source**  Electronic Health Records - SLaM CRIS database | **Sample size**  2026 patients with first episode psychosis, including 100 patients diagnosed with bipolar disorder. | **Clinical Relevance Proposed by Author**  Poor insight has an important prognostic role and measures to improve insight may bring important benefits at the service level as well as to an individual's course of illness. | **Journal**  BMJ Open | **Ethical Considerations**  Oxford C Research Ethics Committee, reference 08/H0606/71+5.  The SLaM Biomedical Research Centre Case Register used in the study was developed with extensive PPI and is over-seen by committees that include service user and general public representatives. | **Is the dataset publicly available?**  CRIS database access. | **Is the code available for download?**  Not reported |
| **Focus:** This study sought to extract descriptions of insight from text fields to see if recorded poor insight early after clinical presentation predicted subsequent service use. | | | | | | | | | |
| **Method:** TextHunter annotation software used to create training and test corpora classifying mentions of insight in the clinical record to train a supervised machine learning algorithm to recognize this automatically across the wider sample. The algorithm was then used to locate recorded poor insight from case records within 1 month either side from the date a patient was accepted into an early intervention service. | | | | | | | | | |
| **Key Findings:** The algorithm identified 826 patients out of the original 2026 as having poor insight. 61% of the bipolar patients within the cohort had at least one recording of poor insight within a month of being accepted to an early intervention service, and poor insight was most common in the bipolar disorder cohort.  Higher numbers of hospitalization episodes , higher odds of legally enforced hospitalizations , higher numbers of unique antipsychotics and higher numbers of inpatient days were all significantly associated with poor insight as measured at 12, 24, 36, 48 and 60 months. | | | | | | | | | |
| **Objective (Annotated by reviewer through inductive analysis):** Using EHRs to measure health outcomes^a^ | | | | | | | | | |

| **Practical issues in developing semantic frameworks for the analysis of verbal fluency data: A Norwegian data case study (Rosenstein et al.)** [58] | | | | | | | | | | | | | | | | | |
| --- | --- | --- | --- | --- | --- | --- | --- | --- | --- | --- | --- | --- | --- | --- | --- | --- | --- |
| **First Author Discipline**  Computer science | **Study Context**  Schizophrenia and bipolar disorder. | | **Diagnosis confirmed (where relevant) e.g. by self-report**  Participants with a confirmed diagnosis of bipolar disorder from Oslo University Hospital | | **Data Source**  Electronic Health Records - SLaM CRIS database | | **Sample size**  100 case participants.  75 patients with bipolar disorder.  50 control. | | **Clinical Relevance Proposed by Author**  The results demonstrate that semantic structures are affected by cortical disorders and can be a key feature of the diagnosis of bipolar disorder. | | **Journal**  Proceedings of the 2nd Workshop on Computational Linguistics and Clinical Psychology: From Linguistic Signal to Clinical Reality, pages 124–133, Denver, Colorado, June 5, 2015 | | **Ethical Considerations**  Not discussed | **Is the dataset publicly available?**  Not reported | | | **Is the code available for download?**  Not reported |
| **Focus:** Provide a framework for measuring how semantic structure is affected by cortical disorders. | | | | | | | | | | | | | | | | | |
| **Method:** Verbal fluency task conducted with participants where they produce a series of words in response to a cue within a time limit. The resulting semantic relations from the task were compared to latent semantic analysis (LSA) which built up a semantic representation of words from a large newspaper corpus. | | | | | | | | | | | | | | | | | |
| **Key Findings:** Three measures for analysis: mean number of words per diagnostic group, semantic coherence between pairs of words (mean cosine), and cluster measure (num. clusters/ num words.) Control group results consistent with normative results reported by others, where mean animal list word length is 23.5.  Direction of change is consistent among three measures, number of words decreases from control to BD to schizophrenia and semantic coherence between pairs of words also drops in that order. Cluster fraction moves in the expected opposite direction indication more diverse topics. | | | | | | | | | | | | | | | | | |
| Control group: num words: 23.92 mean cos: 0.172 cluster frac: 0.736 | | Bipolar disorder: num words: 20.12 mean cos: 0.151 cluster frac: 0.778 | |  | |  | |  | |  | |  | | |  |  | |
| **Objective (Annotated by reviewer through inductive analysis):** Characterizing the language of bipolar disorder | | | | | | | | | | | | | | | | | |

| **A Framework for Classifying Online Mental Health-Related Communities with an Interest in Depression (Saha et al.)** [59] | | | | | | | | | | | | | | | | | |
| --- | --- | --- | --- | --- | --- | --- | --- | --- | --- | --- | --- | --- | --- | --- | --- | --- | --- |
| **First Author Discipline**  Data science | **Study Context**  12 interest categories: Abuse, anorexia, anxiety, bipolar disorder, cutting, death, drugs, eating disorders, insomnia, pain, self-injury, and suicide. | | **Diagnosis confirmed (where relevant) e.g. by self-report**  No | | **Data Source**  LiveJournal - Personal blogs | **Sample size**  Bipolar disorder class formed 14% of the dataset, around 86k posts and 11k users.  Total dataset 620,000 posts made by 80,000 users. | | **Clinical Relevance Proposed by Author**  The research can contribute to the screening and monitoring of online health communities. | | | **Journal**  IEEE Journal of Biomedical and Health Informatics | | **Ethical Considerations**  Not discussed | | **Is the dataset publicly available?**  Extracted features provided at a link, link no longer works. | | **Is the code available for download?**  Not reported |
| **Focus:** Joint learning framework to learn about co-morbid mental health conditions. | | | | | | | | | | | | | | | | | |
| **Method:**  2 feature sets were extracted during the experiments: 1. language style using LIWC 2. topics using LDA - number of topics set to 50   Using a machine learning technique, a joint modeling framework was formulated in order to classify mental health-related co-occurring online communities from these features. The model is trained using the topic features as an input and the 12 interest categories as outcomes, then the same process is repeated instead using the LIWC features as input. Finally both features are combined into a single model, and single task logistic regression was compared against multi-task learning. 70:20:10 split for training, test and validation. | | | | | | | | | | | | | | | | | |
| **Key Findings:** Using only one feature set, the multi-task learning performs better than the single task learning, but when combining both types of features the models perform the same, and the latent topics are found to have a greater predictive power than linguistic features. Results indicate that the distinct topics and linguistic styles have a strong predictive power to classify mental health-related communities with an interest in depression. | | | | | | | | | | | | | | | | | |
| Predictive topic features for bipolar disorder: cutting blood scars, depression meds anxiety disorder, heart pain inside  Predictive LIWC features for bipolar disorder: sad, death (neg emo) | |  | | Bipolar disorder results AUC:  STL LIWC - 0.789 STL Topics - 0.874 STL LIWC + TOPICS - 0.899 MTL LIWC - 0.817  MTL TOPICS - 0.887 MTL LIWC + TOPICS - 0.899 | | |  | |  |  | |  | |  | |  | |
| **Objective (Annotated by reviewer through inductive analysis):** Prediction and classification | | | | | | | | | | | | | | | | | |

| **MIDAS: Mental illness detection and analysis via social media (Saravia et al.)** [60] | | | | | | | | | |
| --- | --- | --- | --- | --- | --- | --- | --- | --- | --- |
| **First Author Discipline**  Natural language processing and affective computing | **Study Context**  Bipolar disorder and borderline personality disorder. | **Diagnosis confirmed (where relevant) e.g. by self-report**  Relied on presence of specific keywords in Twitter bios from mental health community portals | **Data Source**  Twitter | **Sample size**  481 total user accounts.  278 bipolar disorder users.  548 control users. | **Clinical Relevance Proposed by Author**  Tools such as MIDAS may be able to assist in discovering vulnerable geographic regions and the timely distribution of treatment. | **Journal**  2016 IEEE/ACM International Conference on Advances in Social Networks Analysis and Mining (ASONAM) | **Ethical Considerations**  Identity of the samples used in the demonstration not disclosed and referred to as sample A and sample B. | **Is the dataset publicly available?**  Not reported | **Is the code available for download?**  Not reported |
| **Focus:** Predictive model for mental disorders based on Twitter data. | | | | | | | | | |
| **Method:**  Two sets of features extracted: (TFIDF) - unigrams and bigrams - and Pattern of Life features  -Models trained to produce statistics that are leveraged to build an online visualization tool to analyze characteristics of Twitter users  Pattern of life features include: -Age and gender  -Polarity features - each tweet labelled as positive, negative or neutral, and polarity of each user also calculated  -Social features i.e. Tweeting frequency, mention ratio, frequent mentions, unique mentions   Separate classifiers were trained for each disorder, 10 fold cross validation, random forest classifier. | | | | | | | | | |
| **Key Findings:**  Bipolar disorder classification: TF IDF features - 96 % precision Pattern of life features - 91% precision | | | | | | | | | |
| **Objective (Annotated by reviewer through inductive analysis):** Prediction and classification | | | | | | | | | |

| **Not just depressed: Bipolar disorder prediction on Reddit (Sekulić et al.)** [61] | | | | | | | | | |
| --- | --- | --- | --- | --- | --- | --- | --- | --- | --- |
| **First Author Discipline**  Computer science and natural language processing | **Study Context**  Bipolar disorder. | **Diagnosis confirmed (where relevant) e.g. by self-report**  Self-reported diagnosis (searching the comments of bipolar subreddits for patterns such as 'I was diagnosed with bipolar') | **Data Source**  Reddit | **Sample size**  3488 bipolar disorder users and 3931 control users. | **Clinical Relevance Proposed by Author**  There is a need to develop systems which are capable of early detection of suicide due to the high incidence of death by suicide in bipolar disorder. | **Journal**  Proceedings of the 9th Workshop on Computational Approaches to Subjectivity, Sentiment and Social Media Analysis, pages 72–78 Brussels, Belgium, October 31, 2018 | **Ethical Considerations**  Not discussed | **Is the dataset publicly available?**  Not reported | **Is the code available for download?**  Not reported |
| **Focus:** Bipolar disorder prediction from self-reported diagnosis. | | | | | | | | | |
| **Method:**  For each user 1) psycholinguistic features, 2) lexical features and 3) reddit user features were extracted. (1) LIWC used for psycholinguistic analysis including 93 features. Stanford Empath also used. (2) Lexical features come from TFIDF weighted BoW stemmed using NLTK. (3) Reddit features model users' interaction patterns  Bipolar disorder prediction framed as a binary classification task using 3 classifiers, SVM, LR and RF. | | | | | | | | | |
| **Key Findings:**  Personal pronouns more often used by bipolar disorder users, as well as more words associated with feelings, health and biological processes. Significant use of 'anxiety' words. Bipolar disorder users demonstrate more valence over time, possibly illustrating the alternation of manic-depressive moods.   Highest performing classifier was random forest with an F1 score of 0.86, and the best models used all three features or TFIDF alone. | | | | | | | | | |
| **Objective (Annotated by reviewer through inductive analysis):** Prediction and classification | | | | | | | | | |

| **Adapting deep learning methods for mental health prediction on social media (Sekulić & Strube 2020)** [62] | | | | | | | | | |
| --- | --- | --- | --- | --- | --- | --- | --- | --- | --- |
| **First Author Discipline**  Computer science and natural language processing | **Study Context**  Depression, ADHD, anxiety, bipolar disorder, PTSD, autism, OCD, schizophrenia, eating disorder. | **Diagnosis confirmed (where relevant) e.g. by self-report**  Self-reported diagnosis (using patterns to match diagnosis statements e.g. 'I was diagnosed with BD today') | **Data Source**  Reddit | **Sample size**  Total 20,406 diagnosed users and 335,952 matched controls.  6434 self-reported diagnosed users with bipolar disorder, 575k posts, 157.6 posts per user. | **Clinical Relevance Proposed by Author**  In-depth text analysis can contribute to deeper understanding of illnesses and provide means for their early detection. | **Journal**  Proceedings of the 2019 EMNLP Workshop W-NUT: The 5th Workshop on Noisy User-generated Text, pages 322–327 Hong Kong, Nov 4, 2019 | **Ethical Considerations**  Comply with the data usage agreement and do not identify the users in the dataset or link them with other information. | **Is the dataset publicly available?**  Available through a data usage agreement | **Is the code available for download?**  Not reported |
| **Focus:** Detecting social media user status through deep learning models, to predict whether user belongs to control or diagnosed group. | | | | | | | | | |
| **Method:** A hierarchical attention network is used in this study. A HAN consists of a word sequence encoder, a word-level attention layer, a sentence encoder and a sentence level attention layer - ultimately yielding a document representation. In this work, one user is modelled as a document, using the posts as sequences of sentences. TFIDF weighted BoW features used as features, where users' posts are concatenated and lower cased.  Binary classification task predicting if a user has one of the 9 different disorders. | | | | | | | | | |
| **Key Findings:**  HAN outperforms the benchmarks previously set for four of the disorders, but the results suggest that neural models are data hungry as higher F1 scores are achieved for the conditions with more data.  The best performing classification model for the bipolar disorder dataset is the HAN model, with 67.42 F1 measure. | | | | | | | | | |
| **Objective (Annotated by reviewer through inductive analysis):** Prediction and classification | | | | | | | | | |

| **Predicting user emotional tone in mental disorder online communities (Silveira et al.)** [15] | | | | | | | | | | | | | | | | | |
| --- | --- | --- | --- | --- | --- | --- | --- | --- | --- | --- | --- | --- | --- | --- | --- | --- | --- |
| **First Author Discipline**  Computer science | **Study Context**  Depression, suicide, anxiety and bipolar disorder. | **Diagnosis confirmed (where relevant) e.g. by self-report**  No | | **Data Source**  Reddit | **Sample size**  Total dataset: 141,835 threads, 154,114 unique users.  15,825 threads within the bipolar disorder class. Average of 134 comments per thread. 11,363 unique users. | | **Clinical Relevance Proposed by Author**  This type of work could provide supplementary data to clinical care, helping to provide timely interventions. | | | | **Journal**  Journal of Future Generation Computer Systems | | **Ethical Considerations**  Models do not consider any user features so can be implemented without harming privacy. | | **Is the dataset publicly available?**  Code to scrape the same datasets: https://github.com/HenrySilvaCS/SentiMentalHealth | **Is the code available for download?**  https://github.com/HenrySilvaCS/SentiMentalHealth | |
| **Focus:** To analyze how interactions affect people with mental disorders who use online social networks e.g. are people who write initially negative posts happier after receiving social support? | | | | | | | | | | | | | | | | | |
| **Method:**  Uses the emotional tone of the first post and the comments that are made on it to predict the emotional tone of the last comment made by the thread author.  VADER compound score used to measure emotional tone (computes 4 variables: positive, negative, neutral and compound), DistilBERT embeddings used as model input.  Recurrent neural network using weighted L1 loss as the loss function. | | | | | | | | | | | | | | | | | |
| **Key Findings:**  Users in the bipolar disorder subreddit are the most active, writing at least twice as often as users in other communities - showing a stronger engagement in that subreddit. The results show that in general, emotional states improve after interactions on a thread, the emotional tone of the final post is more positive than the emotional tone of the initial post, and the comments on the post are more positive than the post that initiated the thread. The model for all subreddits outperforms all of the baselines by at least 12.9 %.  Weighted L1 loss for bipolar disorder class: | | | | | | | | | | | | | | | | | |
| Baseline - mean of sequences: .489 Baseline - emotional tone last comment: .513 Baseline - XGBoost pooling: .500 | | | Model (all subreddits): .401 Model (bipolar subreddit): .491 | | |  | |  |  |  | |  | |  | | |  |
| **Objective (Annotated by reviewer through inductive analysis):** Prediction and classification | | | | | | | | | | | | | | | | | |

| **Predicting future mental illness from social media: A big-data approach (Thorstad & Wolff 2019)** [63] | | | | | | | | | |
| --- | --- | --- | --- | --- | --- | --- | --- | --- | --- |
| **First Author Discipline**  Data science | **Study Context**  ADHD, bipolar disorder, anxiety, depression. | **Diagnosis confirmed (where relevant) e.g. by self-report**  No | **Data Source**  Reddit | **Sample size**  3 studies:  Study 1: 56,009 posts from bipolar subreddit, 224,036 total posts Study 2: 6109 users per disorder, 24,436 total users  Study 3: 4,513 users per disorder, 18,052 total users | **Clinical Relevance Proposed by Author**  Classifiers could be built to identify those at risk of developing mental illnesses through screening language. | **Journal**  Journal of Behavior Research Methods | **Ethical Considerations**  Discussion around dual-use dilemma and authors suggest that people could be notified that their casual online comments may be mined. | **Is the dataset publicly available?**  Data available upon request | **Is the code available for download?**  Not reported |
| **Focus:** To understand whether everyday language contains enough signals to predict the future occurrence of mental illness. | | | | | | | | | |
| **Method:**  3 x studies within paper  1. Train a model to identify the mental illness from the subreddit from which it was drawn.  -Data scraped from reddit using the reddit API -Pre-processing to remove explicit mentions to clinical disorders and transforming text into TFIDF vectors using unigrams in scikit learn. -ML model trained using words in the post as input, and to output the subreddit the post was made to. Logistic regression model - 4 separate binary classifiers for each condition, and the output of the model is the label assigned with the highest probability by any of the four binary classifiers. Chance performance is 25%. -Clustering analysis performed to observe the most predictive features of the model.   2. Determine whether nonclinical contexts still reveal information about mental health -For all of the individuals who posted to a clinical subreddit in study 1, all of their posts on different subreddits were also downloaded, and the same logistic regression models were trained -Dataset was under sampled for 6,109 users per disorder -Data pre-processing and ML architecture identical to study 1  3. Future prediction - use everyday language to predict future occurrence of mental illness from a non-clinical subreddit -Downloaded all of the posts of any user who posted on the clinical subreddits -Found the first date that the individual posted to a clinical subreddit and then eliminated any posts written on or after that date -Same logistic regression model trained as in study 1 and 2 | | | | | | | | | |
| **Key Findings:**  Study 1: F1 score 0.75 for predicting bipolar disorder vs. other clinical subreddits. Study 2:  F1 score 0.34 for predicting bipolar disorder from nonclinical subreddits, above chance.  Study 3:  F1 0.37 for predicting future mental disorder from nonclinical subreddits for bipolar disorder, above chance. | | | | | | | | | |
| **Objective (Annotated by reviewer through inductive analysis):** Prediction and classification | | | | | | | | | |

| **Predicting mental conditions based on "history of present illness" in psychiatric notes with deep neural networks (Tran and Kavuluru)** [64] | | | | | | | | | |
| --- | --- | --- | --- | --- | --- | --- | --- | --- | --- |
| **First Author Discipline**  Computer science | **Study Context**  Depression, bipolar disorder, psychosis, panic disorder, anxiety spectrum disorders, obsessive compulsive disorders (OCD), attention deficit hyperactivity disorder (ADHD),post-traumatic stress disorder (PTSD), eating disorders, dementia, and complicated grief. | **Diagnosis confirmed (where relevant) e.g. by self-report**  Diagnosis confirmed from medical notes | **Data Source**  Electronic Health Records | **Sample size**  986 out of the 1000 notes used as final dataset.  33% of the dataset labelled with bipolar disorder (326 notes). | **Clinical Relevance Proposed by Author**  The incorporation of the attention mechanism provides 'informativeness weights' which could help physicians to make more informed decisions and expedite tasks. | **Journal**  Journal of biomedical informatics | **Ethical Considerations**  Not discussed | **Is the dataset publicly available?**  Potentially available upon request, prior registration for task required | **Is the code available for download?**  Not reported |
| **Focus:** Predict a set of common mental health conditions based on the short textual description of the patient's history of present illness. | | | | | | | | | |
| **Method:**  -Pre-processing of dataset to fix line breaks etc.  -Deep neural network to predict a binary yes/ no for each mental health condition (multi-label text classification problem). Predictions made on all 11 psychiatric labels simultaneously to account for label correlation. Tested with both a CNN and an RNN with attention mechanism.  -Neural word embeddings used from PubMed extracts -Full note used as input to the model  -Also tested a number of other classifiers for baseline performance | | | | | | | | | |
| **Key Findings:**  -The short history of present illness segment in a psychiatric evaluation note can be used as a good predictor for a few psychiatric conditions.  Bipolar disorder best performing classifiers:  Precision: Named entity recognition model (based on manually curated entities) 79.9% Recall: ReHAN 54% F: CNN+Threshold 56.7% | | | | | | | | | |
| **Objective (Annotated by reviewer through inductive analysis):** Prediction and classification | | | | | | | | | |

| **Evaluation of Smoking Status Identification Using Electronic Health Records and Open-Text Information in a Large Mental Health Case Register (Wu et al.)** [65] | | | | | | | | | |
| --- | --- | --- | --- | --- | --- | --- | --- | --- | --- |
| **First Author Discipline**  Background in Psychiatric Epidemiology, PhD in suicide prevention | **Study Context**  Bipolar disorder and schizophrenia. | **Diagnosis confirmed (where relevant) e.g. by self-report**  Diagnosis confirmed from medical notes | **Data Source**  Electronic Health Records - SLaM CRIS database | **Sample size**  195 people with bipolar disorder who had been receiving active healthcare for at least 12 months identified as having a smoking status recorded in their health records. 955 people in total from the population identified as having a smoking status, out of 1,555 people. | **Clinical Relevance Proposed by Author**  EHRs can provide information on smoking status for people living with an SMI, which could help to improve the assessment of smoking behavior and develop cessation strategies. | **Journal**  PLoS ONE | **Ethical Considerations**  The Case Register study received ethical approval as an anonymized data resource for secondary analyses by Oxfordshire REC C in 2008 (reference number 08/H0606/71). Individual consent was not obtained for this study because data had been effectively anonymized by CRIS prior to researcher access. | **Is the dataset publicly available?**  Upon application | **Is the code available for download?**  https://sourceforge.net/projects/crisiesmoking/ |
| **Focus:** Investigate smoking prevalence and factors influencing this in people receiving mental healthcare. | | | | | | | | | |
| **Method:**  Open text fields within health records interrogated using GATE. CRIS-IE smoking application developed to identify different smoker statuses, uses shallow parsing rule-based approach based on keywords. Rules developed based on iterative process of manual gold-standard annotation followed by comparison with results generated at each stage. Process repeated until precision of 93% automatic annotations was achieved.   Algorithm process: -Tokenization/ sentence splitting -PoS tagging -Keyword lookup -Co-referencing -Rule-based detection -Data extraction | | | | | | | | | |
| **Key Findings:**  Using structured info alone, 180 (11.6%) records were retrieved with any information related to smoking, but when adding the algorithm for the unstructured fields, this increased to 995 (64%). 593 of these people were smokers, and 110 of this sample were bipolar. Patients with schizophrenia and schizoaffective disorder had higher smoking prevalence than those with bipolar disorder, which is similar to findings from other studies. | | | | | | | | | |
| **Objective (Annotated by reviewer through inductive analysis):** Using EHRs to measure health outcomes^a^ | | | | | | | | | |

| **Semantic network analysis for understanding user experiences of bipolar and depressive disorders on Reddit (Yoo et al.)** [66] | | | | | | | | | |
| --- | --- | --- | --- | --- | --- | --- | --- | --- | --- |
| **First Author Discipline**  Interaction science | **Study Context**  Bipolar disorder and depressive disorders. | **Diagnosis confirmed (where relevant) e.g. by self-report**  No | **Data Source**  Reddit | **Sample size**  2443 posts in bipolar disorder class.  5409 posts in total dataset. | **Clinical Relevance Proposed by Author**  Better understanding of the user experience could help to avoid mis-diagnosis. | **Journal**  Journal of Information Processing & Management | **Ethical Considerations**  Not discussed | **Is the dataset publicly available?**  Not reported | **Is the code available for download?**  Not reported |
| **Focus:** Semantic network analysis to understand how people with bipolar disorder share their experiences and feelings online. | | | | | | | | | |
| **Method:**  Data collected from subreddits before pre-processing using NLTK. TFIDF matrices then created for the semantic network (which constructs a co-occurrence matrix and network based on the words in texts to discover relational properties of words). Significant words in network identified by degree and eigenvector centralities. Clusters and semantic traits in network identified and LIWC also used for further analysis. | | | | | | | | | |
| **Key Findings:**  The bipolar disorder community network had 134 nodes and 10894 ties with 10 clusters. The clusters were:  -Cost of suffering and emotion -Sleeping problems, night episodes and mood swings -Medication type and side effects -Manic/depressive episodes -Bipolar related mental illness -Time related words -Negative feelings and suicidal thoughts -School issues -Symptoms and feelings -Treatment and after bipolar diagnosis   LIWC analysis showed more negative expressions and more formal and logical expressions used by people on the bipolar subreddit. | | | | | | | | | |
| **Objective (Annotated by reviewer through inductive analysis):** Characterizing the language of bipolar disorder | | | | | | | | | |

^a^ This paper was not included in the objective of prediction and classification because the classification itself was not specific to bipolar disorder.

Table S2. Machine learning methods for the articles which perform prediction/classification

Table S3.Machine learning features for the articles which perform prediction/classification

| **Characteristics** | **Number of articles (n = 25)** | **Corresponding Authors** |
| --- | --- | --- |
|  |  |  |
| **Algorithm Type** |  |  |
| Machine Learning | 19 | [13,16,17,34,36–42,44,48,49,51,59–61,63] |
| Deep Learning | 13 | [14,15,34,37,41–44,48,49,54,62,64] |
|  |  |  |
| **Learning** |  |  |
| Single Task e.g. binary classification | 21 | [13,14,16,34,36–42,44,48,49,51,59–63,17] |
| Multi-Task e.g. multi-class/ multi-label classification | 11 | [14,34,37,41,43,44,48,54,59,63,64] |
| Regression Task | 2 | [13,15] |
|  |  |  |
| **Classification Method** |  |  |
| **Deep Learning Methods** |  |  |
| Feed Forward Neural Network | 2 | [34,41] |
| Convolutional Neural Network (CNN) | 7 | [14,37,41,43,44,49,64] |
| Recurrent Neural Network (RNN) | 3 | [15,43,64] |
| Linear Network | 1 | [14] |
| Supervised fastText (Neural net model) | 1 | [37] |
| Hierarchical Attention Network (HAN) | 2 | [14,62] |
| BERT model (Bidirectional Encoder Representations from Transformers) | 3 | [14,42,54] |
| BERT-ATT (BERT attention based classifier) | 1 | [48] |
| RoBERTa (Robustly Optimized BERT Pretraining Approach) | 1 | [54] |
| ELMo | 1 | [42] |
| Universal Sentence Encoder (USE) | 1 | [42] |
| REALM (Retrieval-Augmented Language Model) | 1 | [48] |
| LSTM (Long Short-Term Memory) | 2 | [44,54] |
| Pattern Attention | 1 | [42] |
| Attention Mechanism Incorporated | 6 | [14,43,44,48,62,64] |
|  |  |  |
| **Machine Learning Methods** |  |  |
| Log Linear Classifier | 2 | [38,39] |
| Logistic Regression | 9 | [13,17,34,37,41,48,59,61,63] |
| Random Forest | 5 | [17,36,42,60,61] |
| Support Vector Machine (SVM) | 4 | [17,41,61,64] |
| Naïve Bayes | 2 | [17,40] |
| Decision Trees | 2 | [17,51] |
| XGBoost (extreme gradient boosting classifier) | 3 | [37,49,51] |
| SGD (Stochastic gradient descent linear classifier) | 1 | [51] |
| ET (extra trees classifier) | 1 | [51] |
| LASSO (least absolute shrinkage and selection operator) | 2 | [13,16] |
| Named Entity Recognition (NER) | 1 | [64] |
| Inference Model | 1 | [44] |

| **Features** | **Number of articles (n = 25)** | **Corresponding Authors** |
| --- | --- | --- |
|  |  |  |
| CLM (Character *n-*gram model) | 5 | [34,38,39,44,62] |
| ULM (Unigram n-gram model)/ Bag of Words | 3 | [38,41,64] |
| TFIDF (Term frequency-inverse document frequency) | 9 | [36,37,41,42,49,60–63] |
| LIWC (Linguistic Inquiry and Word Count) | 11 | [16,17,36–39,42,48,51,59,61] |
| Pattern of Life Features (the emotional patterns and behavioral tendencies of users measured by polarity, emotion and social interactions) | 4 | [17,36,38,60] |
| EMOTIVE feature set | 1 | [17] |
| BERT Embeddings | 3 | [14,48,54] |
| RoBERTa Embeddings | 1 | [54] |
| DistilBERT embeddings | 1 | [15] |
| GloVe Embeddings | 2 | [14,62] |
| Word2vec Embeddings | 3 | [14,49,64] |
| Word Embeddings (not specified) | 4 | [41,43,44,54] |
| Context-aware word representations | 1 | [42] |
| Gender | 1 | [42] |
| Syntactic word graph patterns | 1 | [42] |
| VADER Sentiment analysis | 2 | [15,51] |
| Textacy word and syllable counts | 1 | [51] |
| Textacy readability metrics | 1 | [51] |
| Punctuation count | 1 | [51] |
| Manual lexicons | 2 | [51,64] |
| Topic Modeling (LDA) | 2 | [16,59] |
| Empath features | 1 | [61] |
| Reddit user features (e.g. post-comment ratio, average controversiality) | 1 | [61] |
| Pairwise unigram and bigram dictionaries | 1 | [40] |
| Patient or visit features from health records | 1 | [13] |
| Inferred geolocation labels | 1 | [44] |

Table S4. Data extraction template

| Study Characteristics | Title |  |
| --- | --- | --- |
|  | Author(s) |  |
|  | Lead author contact email |  |
|  | Year |  |
|  | Country of first author(s) |  |
|  | First author discipline |  |
|  |  |  |
| Participant Characteristics | Study context (diagnoses) |  |
|  | Diagnosis confirmed? e.g. by self-report |  |
|  | Sample Size |  |
|  |  |  |
| Design Methods | Main aim/ purpose of the study |  |
|  | Clinical relevance if proposed by author |  |
|  | Data source |  |
|  | Data collection method |  |
|  | NLP pipeline/ tools |  |
|  |  |  |
| Ethics and Reproducibility | Ethical considerations |  |
|  | Is the dataset publicly available? |  |
|  | Is the code available for download? |  |
|  |  |  |
| Results | Qualitative description of results |  |
|  | NLP Method Performance indicator (e.g. accuracy/ precision/ recall/ F1 score) |  |

Figure S1. Full search strategy

| Abbreviations | **ADHD**: Attention Deficit Hyperactivity Disorder; **AUC**: Area Under Curve; **BD**: Bipolar Disorder; **BERT-ATT**: Bidirectional Encoder Representations Attention; **BERT**: Bidirectional Encoder Representations; **BoW**: Bag of Words; **BPD**: Borderline Personality Disorder; **CLM**: Character Language Model; **CNN**: Convolutional Neural Network; **CRIS**: Clinical Record Interactive System; **CV**: Cross Validation; **DistilBERT**: Distillated-BERT; **DT**: Decision Trees; **EHR**: Electronic Health Record; **ELMo**: Embeddings from Language Model; **F score**: Score combining precision and recall; **FPR:** False Positive Rate; **GATE**: General Architecture for Text Engineering; **GloVe**: Global vectors for word representation; **GPU**: Graphics Processing Unit; **HAN**: Hierarchical Attention Network; **IRB**: Institutional Review Board; **L**: Loss; **LASSO**: least absolute shrinkage and selection operator; **LDA**: Latent Dirichlet Allocation; **LIWC**: Linguistic Inquiry and Word Count; **LR**: Logistic Regression; **LSA**: Latent Semantic Analysis; **LSTM:** Long Short Term Memory; **MH:** Mental Health; **MI**: Mental Illness; **ML**: Machine Learning; **MTL**: Multi Task Learning; **NB:** Naive Bayes; **NER**: Named entity recognition; **NLP**: Natural Language Processing; **NLTK**: Natural Language Toolkit; **PoL**: Pattern of Life; **PoS**: Part of Speech; **PPV**: Positive Predictive Value; **PTSD**: Post Traumatic Stress Disorder; **REALM**: Retrieval Augmented Language Model; **ReHAN**: Recurrent Neural Networks with Hierarchical Attention; **RF:** Random Forests; **RNN**: Recurrent Neural Network; **RoBERTa**: Robustly Optimized BERT Pre-training Approach; **ROC**: Receiver Operating Characteristic; **SCID-IV**: Structured Clinical Interview for DSM-IV; **SE**: Standard Error; **SGD**: Stochastic Gradient Descent; **SLaM:** South London and Maudsley ; **SMOG**: Simple Measure of Gobbledygook; **SMOTE**: Synthetic Minority Oversampling Technique; **STL**: Single Task Learning; **SVM**: Support Vector Machine; **TFIDF**: Term Frequency Inverse Document Frequency; **TPR**: True Positive Rate; **ULM**: Unigram Language Model; **USE**: Universal Sentence Encoder; **VADER**: Valence Aware Dictionary for Sentiment Reasoning; **w2v**: word2vec; **XGBoost**: eXtreme Gradient Boosting |
| --- | --- |
